# Quality Control Procedures and Baseline Values for Electroretinography, Perimetry, Color Vision, and Visual Acuity in an International Multicenter Study: Observations from a Safety Trial in Chronic Stable Angina Pectoris

**DOI:** 10.1167/tvst.9.8.38

**Published:** 2020-07-28

**Authors:** Eberhart Zrenner, Graham E. Holder, Ulrich Schiefer, John M. Wild

**Affiliations:** 1 Center for Ophthalmology, University of Tübingen, Tübingen, Germany; 2 Werner Reichardt Center for Integrative Neuroscience (CIN), University of Tübingen, Tübingen, Germany; 3 Moorfields Eye Hospital, London, UK; 4 University College London, Institute of Ophthalmology, London, UK; 5 Competence Center Vision Research, University of Applied Sciences Aalen, Aalen, Germany; 6 College of Biomedical Sciences, Cardiff University, Cardiff, UK

**Keywords:** ERG reference values, multicenter trials, visual field reference data, color vision reference data, quality control

## Abstract

**Purpose:**

To describe quality control procedures and baseline values of electroretinography (ERG), kinetic and static perimetry, color discrimination, and best-corrected visual acuity from a multicenter ocular safety study.

**Methods:**

A multicenter prospective longitudinal randomized placebo-controlled study was conducted at 11 ophthalmic centers that had received certification following training, instruction, and monitoring. ERGs were obtained with the Espion E2 Ganzfeld console, perimetry with the Octopus 101 perimeter, color discrimination with the Lanthony desaturated D15 test, and best-corrected visual acuity with the Early Treatment Diabetic Retinopathy Study chart. Ophthalmic eligibility required satisfactory outcomes for ERG and perimetry by the second or third pre-inclusion attempts, respectively. Quality control for the ERG was undertaken by two central readers.

**Results:**

The mean (SD) age of the 97 individuals was 63.5 (7.9) range, 44–83 years. The overall coefficients of variation (CVs) for the ERG peak times were less than those of the only comparable single-center study. The CV for the mean defect of standard automated perimetry was approximately one-third that of the Ocular Hypertension Treatment Study. With increasing age, ERG peak times and color discrimination Total Error Score increased while ERG amplitudes and isopter area all decreased.

**Conclusions:**

The data illustrate the benefit of identical equipment, stringent on-site instruction and training, quality control, certification, and validation methods. The latter are recommended for planning and conducting multicenter trials using ERG and perimetry to monitor safety and/or efficacy of treatment intervention.

**Translational Relevance:**

Stringent quality control procedures and reliable reference values are indispensable prerequisites for informative clinical trials.

## Introduction

The comparison of a given measure of structural or functional integrity to those from a corresponding and representative distribution of age-corrected normal values has become commonplace in ophthalmic practice. Abnormality is defined, statistically, in terms of the given measure lying beyond the range of corresponding normal values. The likelihood of detecting an abnormality is, therefore, dependent upon the magnitude of the variability associated with the measurement itself and upon the between-individual variability in normal individuals. Similarly, the time point at which a worsening of any given measure can be identified (i.e., a change from baseline) is dependent upon the magnitude of the corresponding within- and between-test variability. The recording conditions for some diagnostic tests (e.g., perimetry and retinal nerve fiber layer thickness assessment by optical coherence tomography) are relatively constant such that a database of normative values can be incorporated into the instrument. However, the outcome for visual electrophysiology is highly influenced by the recording conditions and necessitates a database of normal values for each individual center, acquired using standardized protocols and operating procedures.

Current clinical trials generally recruit participants from multiple centers to minimize the impact of data collection on already overburdened clinics and/or due to the low prevalence of the particular disease. Such a multicenter approach requires standardized acquisition protocols and stringent quality control of the data to obtain the sensitive and specific endpoints required for safety and/or efficacy trials of therapeutic interventions. The quality control for trials in which perimetry is the primary endpoint has long been documented and involves site visits, study manuals, and training programs, accompanied by qualifying assessments and certification procedures for appropriate clinic personnel.^1^^–^^3^ Similar principles have been applied to trials in which visual acuity is the primary endpoint^4^ but have not previously been adopted with similar stringency for multicenter trials involving electroretinography (ERG).

The purpose of the current report is twofold: first, to describe procedures to promote the acquisition of high-quality data (ERG, perimetry, color discrimination, and visual acuity) and, second, to describe the pre-treatment baseline values derived by each investigative technique. The latter is advantageous in that normal values of visual function for the ages of the individuals in the study have received little attention.

## Methods

### Design

The study underlying the pre-treatment baseline values presented here was a multicenter, international, prospective, double-blind, randomized placebo-controlled trial of a selective inhibitor of the cardiac pacemaker current (I*_f_*), ivabradine, in patients with chronic stable angina pectoris who were receiving standard background anti-angina therapy (EudraCT No. 2006-005475-17). The primary objective of the trial, which was requested by the European Medicines Agency, was to “document further the long-term ocular safety of ivabradine.” The primary endpoint was to assess by ERG the expected pharmacologic effect of the I*_f_* inhibitor on the corresponding retinal current, I*_h_*, generated by the hyperpolarization-activated cyclic nucleotide-gated cation channels^5^^–^^7^ and its potential reversibility in ERG. The secondary endpoints were changes in best-corrected visual acuity (BCVA), color vision discrimination, and the visual field.

The study involved 19 cardiology centers and 11 associated ophthalmologic centers distributed across nine countries in Europe, Asia, Australia, and South America. The ophthalmologic centers were specialized in both ERG and perimetry. The study sponsor was advised by an independent Scientific Ophthalmic Safety Committee (SOSC) consisting of two experts in ocular electrophysiology and two experts in perimetry.

### Center Certification

**Figure 1.**
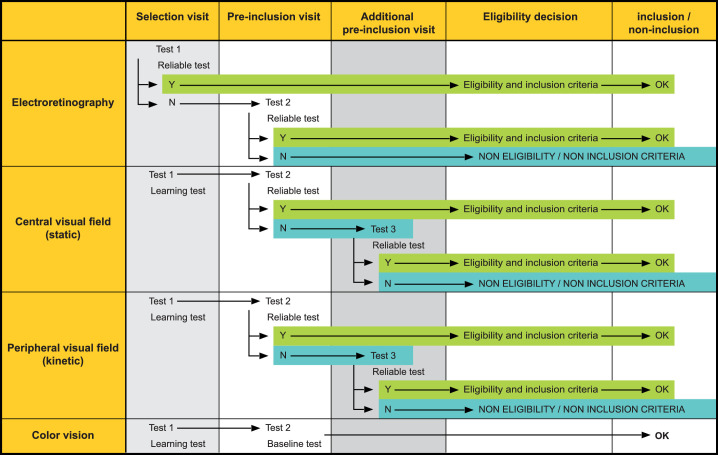
A schematic illustrating the procedure for ophthalmologic eligibility on the basis of a satisfactory/reliable outcome to the quality of ERG and perimetry. Performance with the color vision test was neither an eligibility nor an inclusion criterion. Y = yes/N = no.

The local ophthalmologist investigator and the technicians at each of the 11 centers received 2 days of expert face-to-face hands-on instruction in the study protocol and in the uploading of the data into the central database. At a later date and before the enrollment phase, each ophthalmologic center was required to complete a certification procedure for both ERG and perimetry to the satisfaction of the respective experts of the SOSC who were masked to the center. The certification procedure required technically satisfactory outcomes for each of three ERG recordings, assessed by experts in ERG, as well as three peripheral and three central visual field (VF) examinations, assessed by experts in perimetry (i.e., from nine individuals), together with a successful electronic transmission of the results to the central database (see Annex 1 and 5). If any of the quality criteria were not met, the investigator was given two further opportunities to demonstrate satisfactory competency in the requisite test or tests (Fig. 1).

### Enrollment of Individuals

Potential participants initially attended a pre-inclusion visit for cardiovascular examination by the main investigator, the local cardiologist. The principal cardiac eligibility criteria comprised chronic stable angina pectoris treated with standard background antiangina therapy and a heart rate of at least 60 bpm. There was no age limitation. Medical exclusion criteria comprised renal insufficiency, severe hepatic impairment, severe hypotension, and unstable chronic angina.

Prior to inclusion in the study, the potential individuals then underwent, at the corresponding ophthalmology center and within 10 days of the cardiovascular examination, an ophthalmologic examination including slit-lamp biomicroscopy of the anterior segment, tonometry, ophthalmoscopy, and fundus photography. They subsequently underwent, at the same visit, ERG recording, static and kinetic perimetry, and color vision testing (Fig. 1). Those who failed to produce satisfactory ERG recordings were given one further opportunity, at the first of two pre-inclusion visits undertaken within 5 days of the initial ophthalmologic visit, to produce an acceptable recording. The color vision testing and both types of perimetry were repeated for all potential individuals at the first pre-inclusion visit to reduce the impact of learning effects, particularly in perimetry.^8^ Those who failed to produce a reliable result for perimetry at the first pre-inclusion visit underwent a final attempt, within 5 days, at the second pre-inclusion visit. Eligibility with respect to ERG and perimetry was based upon a reliable performance, verified and validated by an Interactive Voice Response System (IVRS). The latter was an independent structure that was used to ensure a high-quality control system of the correct and validated inclusion criteria and of the study treatment allocation, according to the study protocol. Investigators were required to electronically transmit the relevant information for a given individual to the IVRS, which then verified the qualitative electrophysiologic and the quantitative visual field inclusion criteria and delivered a yes/no response as to eligibility. When an individual became eligible, the IVRS then communicated the kit number of the study drug to the main investigator (the cardiologist) at the corresponding center.

The ophthalmic exclusion criteria comprised a BCVA of worse than 0.5 in either eye; previous intraocular surgery (other than longstanding uncomplicated cataract extraction with posterior chamber lens implantation); spherical ametropia exceeding ±5.00 diopters, respectively, in either eye; astigmatism exceeding 3.00 diopters in either eye; intraocular pressure exceeding 25 mm Hg in either eye, if associated with glaucomatous damage to the optic nerve head and/or a visual field defect characteristic of primary open-angle glaucoma; angle-closure glaucoma; proliferative diabetic retinopathy; macular edema; history of unexplained visual field loss; progressive optic nerve, retinal, or choroidal, disease other than that due to age; and concomitant medical therapy known to have an adverse effect on retinal function.

### Visual Function Testing Protocol, Quality Control, Data Management, and Data Flow

BCVA was measured at 4 m using the Early Treatment Diabetic Retinopathy Study (ETDRS) chart (retro-illuminated light box: EVA Tester; STZ Biomed, Tübingen, Germany) and was recorded in decimals. The BCVA was entered into the electronic case report form (e-CRF) as a decimal value and automatically converted into logarithm of the minimum angle of resolution (logMAR) value.

Color vision discrimination was measured with the Lanthony D15 desaturated color vision test (Luneau, Paris, France) performed under a constant illuminance of 270 lux white light (D 65 standard illuminant) within a Standard Light Box (Judge QC; X-Rite Inc., Grand Rapids, MI, USA). The sequence of the caps was entered into software^9^ available at https://www.torok.info/colorvision/d15.htm, which identifies entry errors and automatically calculates the Total Error Score (TES).^10^ The TES was then entered into the e-CRF (for procedure protocol, see Annex 2). The output of the test was also printed as hard copy, which then became a source data sheet to be uploaded into the central database.

Perimetry was undertaken using the Octopus 101 perimeter (Haag-Streit, Köniz, Switzerland). The peripheral visual field was examined by semiautomated kinetic perimetry (VF kinetic; VFk) using the III4e and I3e Goldmann stimuli presented randomly along each 15° meridian at an angular velocity of 3° s^–1^. The blind spot was defined by presentation of the I4e stimulus at 2° s^–1^ starting from its presumed center along each 30° meridian. The area of the blind spot was automatically calculated by the perimeter. Each isopter was corrected for the reaction time of the given individual, which was calculated from each of three reaction time vectors placed within normal regions of the central field^11^ (for procedure protocol, see Annex 3).

The central field was assessed by standard (static) automated perimetry (VF static; VFs) using Program G1 and stimulus size III and a 4-2-1 dB threshold strategy, with the appropriate near correction in situ. The sensitivity at each individual stimulus location at the immediately preceding visit was used as the starting value for the staircase of the subsequent visit to reduce the number of stimulus presentations prior to the first reversal and, therefore, mitigate against the perimetric fatigue effect,^12^ which is associated with a prolonged bracketing procedure. An inappropriate starting value would have induced a greater systematic error than any inaccuracy in the threshold estimate arising from the use of the previous values of sensitivity, particularly as a triple crossing (4-2-1 dB) of threshold was used at each location and at each visit. Furthermore, the presence of within-individual and within- and between-session variation in response further mititates against the impact of any systematic error associated with the derivation of the threshold estimate in this manner, particularly in the presence of more substantial confounders such as the perimetric learning^8^ and fatigue effects.^12^

The peripheral field was assessed before the central field. The right eye was examined before the left eye for each perimetric modality. A 5- to 10-minute rest period was given between the two examinations.

The standard printout from each type of perimetry for each individual at each visit was stored in a web-based electronic database specifically designed for the study. In addition, the database separately stored the mean reaction time, the reaction time–corrected area for each of the two isopters and for the blind spot, the number of incorrect responses to the false-negative and false-positive catch trials, the magnitude of the Mean Defect (MD) index, the BCVA, and the refractive correction. The outcomes in each eye for the peripheral and central field examinations comprised the reaction time–corrected areal extent of each isopter and the MD index, respectively.

Electroretinography was performed binocularly according to the ISCEV standard, applicable at the time of the study,^13^ using Espion E^2^ Electrophysiology Consoles (Diagnosys LLC, Lowell, MA, USA) and single-use Dawson-Trick-Litzkow (DTL) fiber electrodes^14^ (Diagnosys LLC). The electrodes were standardized since the ERG is influenced by the type of electrode. Each site had a supply of individually packed DTL electrodes, and each electrode had a separate serial number. New DTL electrodes were used at each visit for each individual, and the serial number of the given electrode was entered into the e-CRF by the local investigator. The use of the correct electrode type was confirmed by the local monitor of the study and further verified by the sponsor. Pupils were dilated with either 0.5% or 1% tropicamide, as appropriate. The active electrodes were placed at or just above the margin of the lower eyelid to ensure contact with the lower limbus of the cornea. The reference electrodes were on the zygomatic fossae, and the ground electrode was on the central forehead. An impedance of <5 kΩ was required for each electrode. The custom software prevented recording of the ERG until 20 minutes of dark adaptation (DA) had been completed. The DA ERGs comprised the 0.01 cd·s·m^−2^ (interstimulus interval [ISI], 5 seconds) rod ERG (DA 0.01), the 3.0 cd·s·m^−2^ (ISI, 20 seconds) combined rod-cone standard flash ERG (DA 3.0), and the 12 cd·s·m^−2^ (ISI, 20 seconds) strong flash ERG (DA 12.0). The photopic ERGs comprised the light-adapted (LA) 3.0 cd·s·m^−2^ (ISI, 0.5 seconds) standard-flash “cone” ERG (LA 3.0) and the light-adapted 30-Hz flicker ERG (LA 3.0 flicker) and were recorded after a 10-minute software-controlled light adaptation to a background white light of 34 cd·m^−2^ in the Ganzfeld bowl. The recording technician ensured that the eyes remained open throughout the light adaptation period by observation of the eyes via a camera built into the Ganzfeld bowl (detailed description in Annex 4).

**Figure 2.**
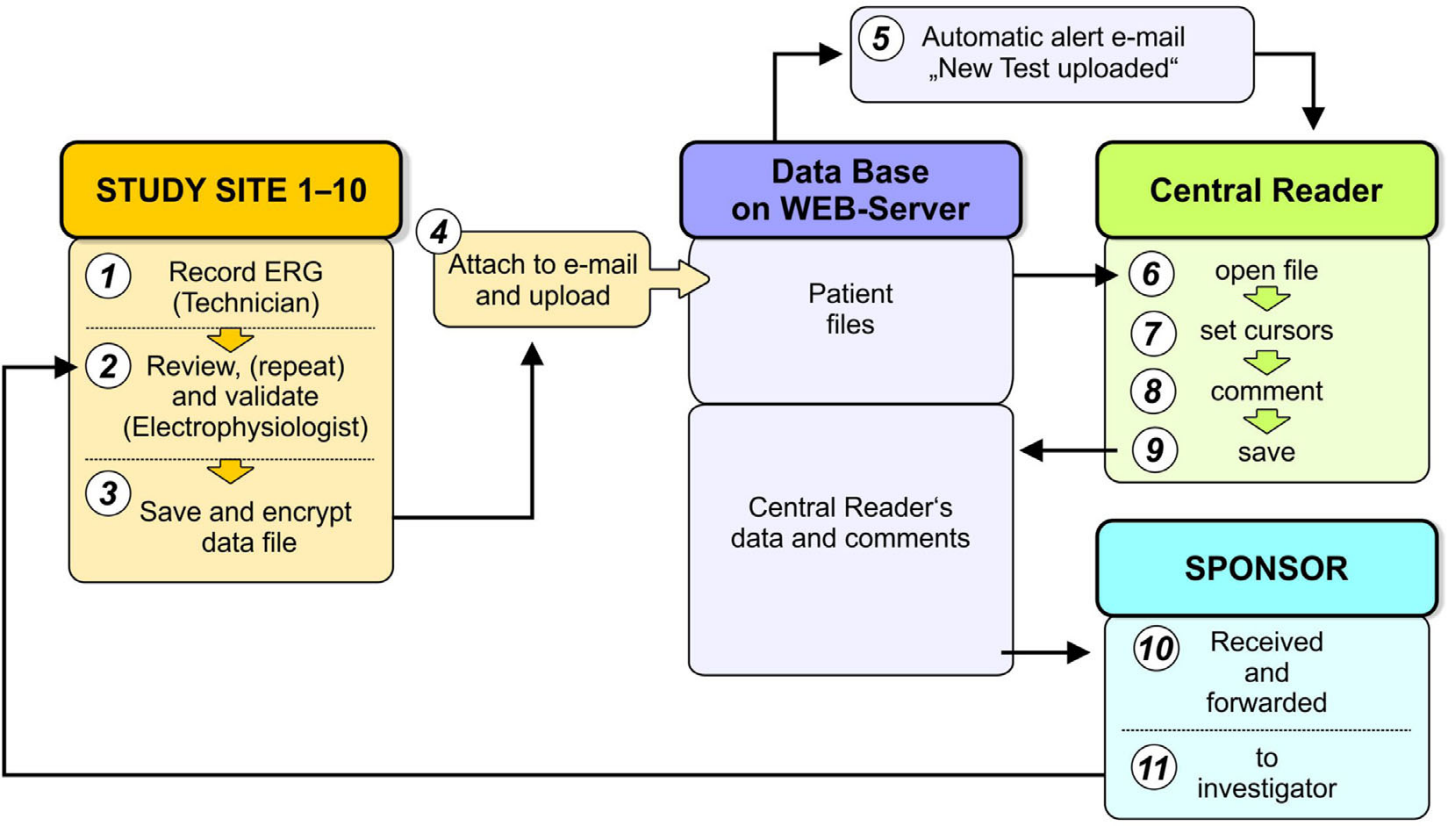
A schematic illustrating the data flow, management, and quality monitoring for the ERG recording. One of the eleven ophthalmologic centers failed to enroll any individuals.

An encrypted file of the outcome of the ERG at each given visit for each individual was created by the Espion Console and uploaded into the central database on a protected web server (Fig. 2). The software and database format were specifically developed for the study (Diagnosys UK Ltd, Cambridge, UK). The central readers in electroretinography (two experienced electrophysiologists from Moorfields Eye Hospital, London, UK) were responsible for reviewing/analyzing the ERG and were masked to the identity of the site that had undertaken the recording.

### Quality Control

The ERG central readers were automatically informed of each upload and had direct access to the web server. The review/analysis of the ERG for any given individual was assigned to either one of the central readers, and the given reader then undertook the review for all of the visits of that individual. The central readers were required, within a period of 5 days, to evaluate and, if necessary, reposition the cursor to each component according to predetermined rules, to save any modifications, and to comment on the technical quality of the recording. The central readers were specifically responsible for inspection of potential artifacts; confirmation of the a- and b-wave peak times and amplitudes derived from the investigators’ cursor positions; determination of abnormal changes in peak times and/or amplitudes; verification of the technical quality of the recordings, including the level of reproducibility; and the recognition of artifacts in the recording. Any change by a central reader was recorded in an audit trail, and the validated values were entered into the database (for procedure protocol, see Annex 5). The comments of the central readers were automatically sent to the sponsor, who then informed the respective ophthalmologic center. The central reader requested a repeat examination if the results were technically unsatisfactory; however, the final decision was the responsibility of the given local ophthalmologic investigator.

The quality criteria for both types of perimetry comprised adherence to the perimetric protocol. For semi-automated kinetic perimetry, the quality control also comprised verification of the accurate localization of the blind spot as an absolute scotoma to the I4e stimulus, the accurate specification of the blind spot area (smaller than 43 square degrees) corrected for an individual reaction time of between 200 and 1500 ms, the presence of ellipsoid-shaped isopters of appropriate area and angular extent, and the absence of any crossing of isopters. The area in square degrees was automatically calculated by the perimeter software. For standard automated perimetry, the quality control comprised verification that the incorrect responses to the false-positive and to the false-negative catch trials were each less than 30%.^2^

The study design did not involve test-retest variability of each variable. Test-retest variability is a function of the magnitude of the “true” value. The determination of the test-retest variability across the entire range of parameters was beyond the scope of the study. Instead, test precision of each ERG recording was controlled throughout the entire study by central readers, and that for perimetry was assessed in terms of the area of the blind spot, the reaction time, and the response to the false-positive and false-negative catch trials.

The e-CRF was uploaded to the study database and backed up by hardcopy. All data modifications were recorded in chronologic order using the audit trail feature of the INFORM and CLINTRIAL software. Monitoring procedures ensured that all data in the e-CRF were complete. A careful masked review of possible data entry errors was performed by double entry of the data, and any issues were resolved prior to the locking of the study database.

### Statistical Analysis

The analysis for each parameter, at the pre-treatment baseline, was undertaken in terms of measures of central tendency and dispersion for the cohort, as a whole, and then in terms of three age groups: <60 years, 60 to 69 years, and ≥70 years.

### Ethical Approval

The study followed the tenets of the Declaration of Helsinki, 1964, and subsequent revisions. The study commenced only after approval had been obtained from the appropriate ethics committee for each participating center. Informed consent was obtained from each individual following an explanation of the purpose of the study and any possible consequences. Protocol amendments to the study protocol were approved by each of the ethics committees.

### Baseline Values

The pre-treatment baseline values, described here, comprised the outcome of the pre-inclusion visit for BCVA and color vision and of the respective visits at which a reliable measure of ERG and each type of perimetry was obtained (Fig. 1).

## Results

### Demographic Characteristics

Of the 190 individuals evaluated by the local cardiologists, 171 were selected for potential enrollment in the study; of these, 141 performed at least one ERG test and 130 performed at least one type of perimetry. A total of 102 individuals satisfactorily completed the ERG and both types of perimetry.

Four of the 102 individuals were subsequently excluded as they were found, prior to enrollment, not to have met either the cardiac or other medical inclusion criteria (three with a heart rate below 60 bpm and one with severe hepatic impairment). In addition, one individual attended for the visual function tests beyond the timeframe specified by the study protocol. The number of individuals undertaking the visual function tests, as well as the number satisfactorily completing the tests, by visit, is given in Table 1. The remaining 97 individuals were enrolled into the study. Of these 97, most (77) attended five centers (28, 18, 16, 9, and 6 individuals, respectively); five additional centers each examined up to 5 individuals. The 11th center failed to enroll any individuals.

**Table 1.** Number of Individuals Undertaking the ERG and Visual Field (VF) Examinations and the Number Achieving a Satisfactory Outcome for Each Investigation at the Selection and at the Two Pre-inclusion Visits

	ERG	ERG	VFs	VFs	VFk	VFk	Satisfactory on
Characteristic	Undertaken	Satisfactory	Undertaken	Satisfactory	Undertaken	Satisfactory	Three Tests
Selection visit	141	88	—	—	—	—	—
Pre-inclusion visit	48	22	130	122	130	118	—
Additional pre-inclusion visit	—	—	11	10	15	11	—
Total number of tests	189	110	141	132	145	129	
Total number of individuals	141	110	130	129	130	125	102
Individuals not passing clinical criteria							5
All criteria satisfactory							97

Color vision abnormalities were not considered an exclusion criterion. VFs means Visual field (static), VFk means Visual field (kinetic).

Most (58.8%) of the 97 individuals were male, and most (88.7%) were Caucasian (4.1% were Asian and 7.2% were of other ethnicity). The mean (SD) age was 63.5 (7.9; range, 44–83; median, 65.0; interquartile range, 58.0–69.0) years. Twenty-nine individuals were aged less than 60 years, 47 between 60 and 69 years, and 21 greater than or equal to 70 years of age.

All females were of nonchildbearing potential due to menopause, hysterectomy, or sterilization with the exception of one individual who received effective contraception. Thirteen individuals were current smokers with a mean (SD) tobacco consumption of 14.8 (9.9) cigarettes per day; 46 were previous smokers.

Nine individuals presented with an age-related cataract, two with age-related macular degeneration, and two with a retinal hemorrhage. One individual reported a history of an unspecified eye injury, the type of which was not documented by the investigator. However, the nature of these disorders was such that each of the individuals met the ophthalmologic eligibility criteria.

All but one individual had relevant events in their medical history. The most frequent were disorders of metabolism/nutrition and of the vascular and cardiac systems, which accounted for 85, 83, and 51 individuals, respectively. Of the 85 individuals with metabolic/nutritional disorders, 45.9% had dyslipidemia, 25.9% had diabetes mellitus (either type 1 or type 2), and 10.6% had hyperlipidemia. None of those with diabetes exhibited diabetic retinopathy. Of the 83 with vascular disorders, 97.6% had hypertension, 76.2% had varicose veins, and 6.1% had peripheral arterial occlusive disease. Of the 51 with cardiac disorders, 35.4% had coronary artery disease and 33.3% had myocardial infarction.

A history of a surgical or medical procedure was reported by 64 of the 97 individuals. The most frequent procedure was coronary angioplasty (13.4%). Antithrombotics had been prescribed for 94.8% of the individuals, lipid-modifying agents for 84.5%, agents acting on the renin-angiotensin system for 74.2%, beta-blockers for 73.2%, and cardiac therapy for 71.1% (mostly organic nitrates, 66.0%).

### Electroretinography

The analysis of the ERG was undertaken on 95 of the 97 individuals. The remaining two individuals were excluded on the basis of unreliable baseline ERGs. Twenty-nine of the 95 individuals were aged less than 60 years, 47 between 60 and 69 years, and 19 greater than or equal to 70 years of age. The male/female ratio of these 95 individuals was 55:40.

The summary statistics for the distributions in each eye at baseline of the dark- and light-adapted ERG a- and b-wave amplitudes and peak times for the 95 individuals are given in Figures 3A and 3B, respectively.

**Figure 3A.**
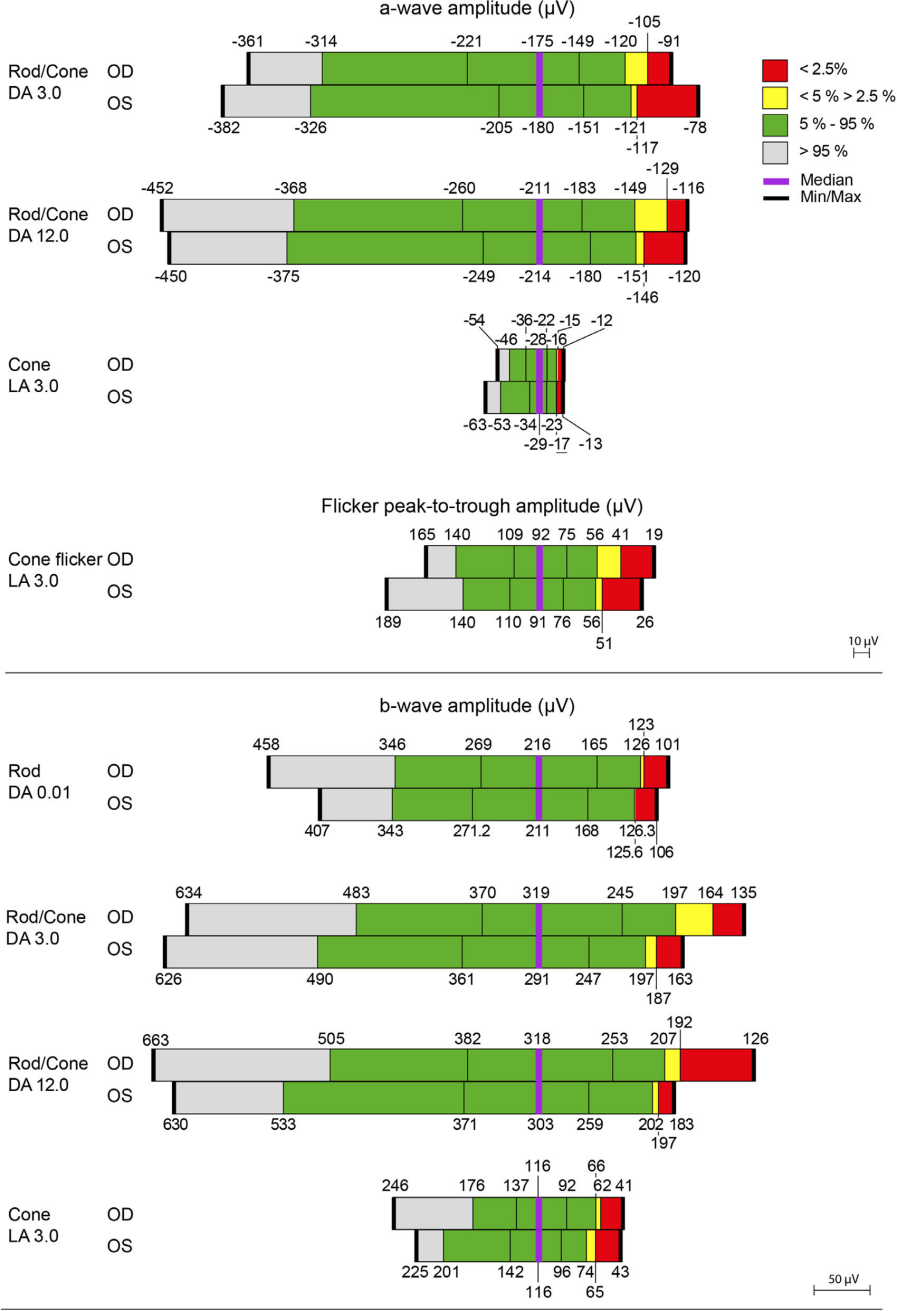
The distribution, for the 95 individuals, of the a-wave (*top*) and b-wave (*bottom*) amplitudes for the right eyes (OD) and left eyes (OS) of the ERG under DA and LA conditions. The values associated with each adaptive state indicate the integrated luminance of the test flash in terms of cd·s·m^−2^. *Gray shading* represents values that are better than the 95th percentile; *green*, values between the 95th and 5th percentiles; *yellow*, values between the 5th and 2.5th percentiles; and *red*, values that are worse than the 97.5th percentile. The two *black vertical lines* within the *green area* delineate the interquartile range. The median is represented by the *purple line* and the maximum and minimum values by the *bold black lines*.

**Figure 3B.**
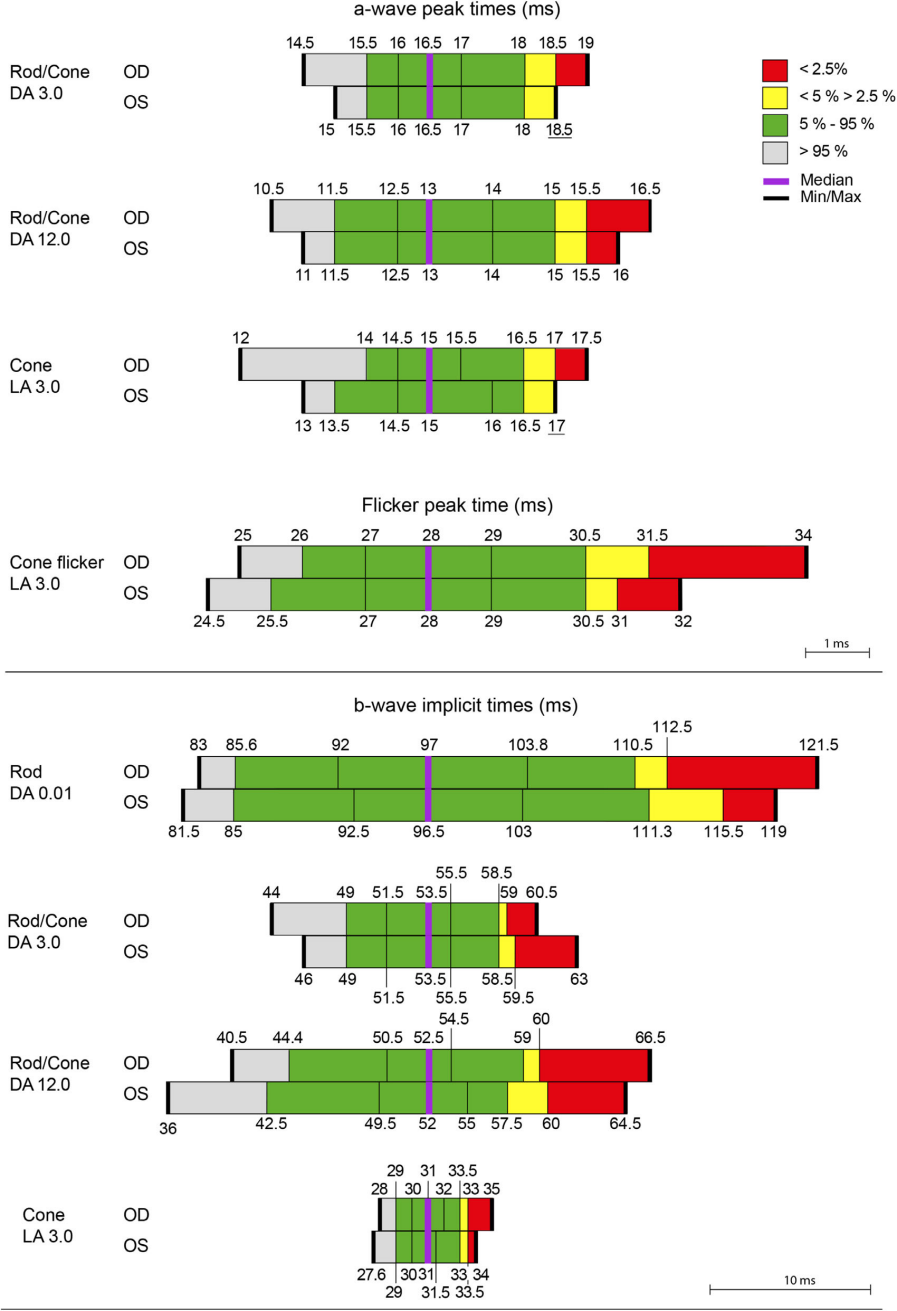
The distribution, for the 95 individuals, of the a-wave (*top*) and b-wave (*bottom*) peak times for the right eyes (OD) and left eyes (OS) of the ERG under DA and LA conditions. The values associated with each adaptive state indicate the integrated luminance of the test flash in terms of cd·s·m^−2^. *Gray shading* represents values that are better than the 95th percentile; *green*, values between the 95th and 5th percentiles; *yellow*, values between the 5th and 2.5th percentiles; and *red*, values that are worse than the 97.5th percentile. The two *black vertical lines* within the *green area* delineate the interquartile range. The median is represented by the *purple line* and the maximum and minimum values by the *bold black lines*. The values for the peak time are given to the nearest half millisecond.

The summary statistics in each eye for the distributions at baseline of the dark- and light-adapted ERG a- and b-wave amplitudes and peak times, averaged across the two eyes for the 95 individuals as a whole and substratified by age group, are given in Table 2 and Table 3, respectively. The amplitudes of all ERGs in each eye were larger for the youngest age group compared to each of the other two age groups. There was no obvious difference in the amplitudes between the two older age groups. The peak times in each eye were shortest for the youngest group.

**Table 2.** Summary Statistics for the Distributions of the ERG Amplitudes, Averaged across the Two Eyes for the 95 Individuals as a Whole and Substratified by Age Group

		Parameter^a^	Amplitude (µV)			
Stimulus^b^			Total (*n* = 95)	<60 y (*n* = 29)	60–69 y (*n* = 47)	≥70 y (*n* = 19)
DA 0.01	b-wave	Mean ± SD	220.7 ± 66.3	240.6 ± 67.0	212.7 ± 61.3	212.4 ± 74.0
		Median [min, max]	206.4 [116.0, 422.2]	228.2 [143.2, 384.1]	201.2 [116.0, 354.7]	202.8 [124.9, 422.2]
		Percentile [5%, 95%]	[135.9, 354.6]	[150.3, 382.2]	[135.3, 339.7]	[124.9, 422.2]
DA 3.0	a-wave	Mean ± SD	−190.3 ± 56.8	−215.1 ± 66.3	−174.47 ± 43.5	−191.5 ± 59.6
		Median [min, max]	−176.8 [−362.5, −84.3]	−197.4 [ −362.5, −122.1]	−168.1 [−300.8, −84.3]	−176.8 [−343.2, −130.5]
		Percentile [5%, 95%]	[−300.8, −122.1]	[−355.1, −130.4]	[−270.2, −121.4]	[−343.2, −130.5]
	b-wave	Mean ± SD	316.9 ± 90.0	345.9 ± 90.2	300.7 ± 82.9	312.7 ± 100.1
		Median [min, max]	306.0 [163.3, 584.4]	330.0 [184.7, 575.2]	284.6 [163.3, 538.5]	306.0 [200.2, 584.4]
		Percentile [5%, 95%]	[202.3, 486.1]	[223.8, 488.0]	[202.3, 468,2]	[200.2, 584.4]
DA 12.0	a-wave	Mean ± SD	−228.1 ± 64.9	−250.7 ± 74.6	−212.7 ± 48.0	−231.5 ± 77.6
		Median [min, max]	−212.4 [−451.2, −123.3]	−221.8 [−406.8, −144.3]	−209.0 [−348.2, −123.3]	−198.9 [−451.2, −167.6]
		Percentile [5%, 95%]	[−359.8, −152.8]	[−377.3, −163.1]	[−320.4, 145.7]	[−451.2, −167.6]
	b-wave	Mean ± SD	327.7 ± 92.6	350.6 ± 95.1	315.4 ± 82.9	323.3 ± 109.1
		Median [min, max]	314.8 [163.4, 622.5]	347.8 [163.4, 579.9]	314.1 [188.2, 532.4]	304.9 [219.6, 622.5]
		Percentile [5%, 95%]	[216.2, 511.8]	[216.2, 511.8]	[214.2, 493.6]	[219.6, 622.5]
LA 3.0	a-wave	Mean ± SD	−29.8 ± 9.0	−32.70 ± 9.4	−28.5 ± 8.2	−28.81 ± 9.4
		Median [min, max]	−28.2 [−54.0, −12.8]	−30.7 [−54.0, −18.8]	−27.9 [−51.3, −12.8]	−26.9 [−52.0, −16.3]
		Percentile [5%, 95%]	[−49.0, −16.9]	[−52.1, −21.2]	[−46.0, −16.9]	[−52.00, −16.3]
	b-wave	Mean ± SD	119.8 ± 35.2	131.8 ± 36.6	114.6 ± 31.5	114.6 ± 39.0
		Median [min, max]	115.2 [46.4, 227.4]	136.8 [67.8, 212.1]	112.3 [46.4, 204.9]	116.0 [68.9, 227.4]
		Percentile [5%, 95%]	[68.9, 187.9]	[35.4, 176.9]	[28.9, 146.6]	[46.1, 131.6]
LA 3.0 Flicker (30 Hz)	Peak to trough	Mean ± SD	93.4 ± 25.8	105.0 ± 30.0	88.27 ± 21.7	88.4 ± 23.9
		Median [min, max]	92.3 [28.9, 176.9]	107.5 [35.4, 176.9]	88.1 [28.9, 146.6]	91.3 [46.1, 131.6]
		Percentile [5%, 95%]	[56.9, 131.6]	[59.9, 169.1]	[59.75, 119.7]	[46.1, 131.6]

The values associated with each adaptive state indicate the integrated luminance of the test flash in terms of cd·s·m^−^^2^.

a Both eyes are averaged.

b DA means dark adapted, LA means light adapted.

**Table 3.** Summary Statistics for the Distributions of the ERG Peak Times, Averaged across the Two Eyes for the 95 Individuals as a Whole and Substratified by Age Group

		Parameter^a^	Peak Time (ms)			
Stimulus^b^			Total (*n* = 95)	<60 y (*n* = 29)	60–69 y (*n* = 47)	≥70 y (*n* = 19)
DA 0.01	b-wave	Mean ± SD	97.6 ± 7.4	95.6 ± 6.0	97.9 ± 7.8	99.9 ± 7.8
		Median [min, max]	96.3 [83.0, 120.0]	94.0 [83.0, 104.5]	96.0 [83.3, 118.5]	100.5 [87.3, 120.0]
		Percentile [5%, 95%]	[85.5, 109.8]	[84.3, 103.8]	[87.5, 111.0]	[87.3, 120.0]
DA 3.0	a-wave	Mean ± SD	16.6 ± 0.8	16.2 ± 0.7	16.7 ± 0.8	17.0 ± 0.8
		Median [min, max]	16.5 [15.0, 18.5]	16.3 [15.0, 17.5]	16.5 [15.3, 18.5]	167.0 [15.8, 18.5]
		Percentile [5%, 95%]	[15.3, 18.0]	[15.0, 17.3]	[15.5, 18.0]	[15.8, 18.5]
	b-wave	Mean ± SD	53.5 ± 2.8	52.9 ± 2.2	53.8 ± 2.5	53.7 ± 4.0
		Median [min, max]	53.5 [45.5, 61.8]	52.8 [49.5, 58.5]	53.8 [46.0, 60.0]	54.5 [45.5, 61.8]
		Percentile [5%, 95%]	[49.0, 57.8]	[49.8, 57.8]	[49.0, 57.3]	[45.5, 61.8]
DA 12.0	a-wave	Mean ± SD	13.2 ± 1.1	12.7 ± 0.9	13.3 ± 1.1	13.6 ± 1.2
		Median [min, max]	13.0 [10.8, 16.0]	12.8 [10.8, 14.8]	13.3 [11.3, 15.8]	13.5 [11.8, 16.0]
		Percentile [5%, 95%]	[11.5, 15.0]	[11.5, 14.3]	[11.5, 15.0]	[11.8, 16.0]
	b-wave	Mean ± SD	51.9 ± 4.0	51.6 ± 3.6	52.0 ± 3.5	52.3 ± 5.7
		Median [min, max]	52.0 [40.5, 65.5]	52.0 [40.5, 61.0]	52.5 [41.8, 60.0]	52.0 [42.0, 65.5]
		Percentile [5%, 95%]	[43.5, 57.5]	[45.0, 55.0]	[45.5, 55.8]	[42.0, 65.5]
LA 3.0	a-wave	Mean ± SD	15.2 ± 0.7	14.9 ± 0.6	15.2 ± 0.7	15.4 ± 0.8
		Median [min, max]	15.3 [13.5, 17.0]	14.8 [13.5, 16.0]	15.3 [13.8, 17.0]	15.3 [13.8, 17.0]
		Percentile [5%, 95%]	[14.0, 16.5]	[13.8, 15.8]	[14.0, 16.5]	[13.8, 17.0]
	b-wave	Mean ± SD	30.9 ± 1.2	30.5 ± 1.1	31.0 ± 1.3	31.3 ± 1.1
		Median [min, max]	30.8 [28.0, 34.5]	30.5 [28.0, 33.0]	31.0 [28.3, 34.5]	31.0 [29.5, 33.3]
		Percentile [5%, 95%]	[29.0, 33.3]	[29.0, 32.5]	[29.0, 33.3]	[29.5, 33.3]
LA 3.0 Flicker (30 Hz)	Peak to trough	Mean ± SD	28.0 ± 1.5	27.6 ± 1.2	28.0 ± 1.6	28.6 ± 1.7
		Median [min, max]	27.8 [24.8, 32.5]	27.3 [25.5, 30.0]	27.8 [24.8, 31.0]	28.5 [26.3, 32.5]
		Percentile [5%, 95%]	[26.0, 30.8]	[26.0, 30.0]	[26.0, 30.5]	[26.3, 32.5]

The values associated with each adaptive state indicate the integrated luminance of the test flash in terms of cd·s·m^−2^.

a Both eyes are averaged.

b DA means dark adapted, LA means light adapted.

### BCVA, Color Vision Discrimination, and Perimetry

**Figure 4.**
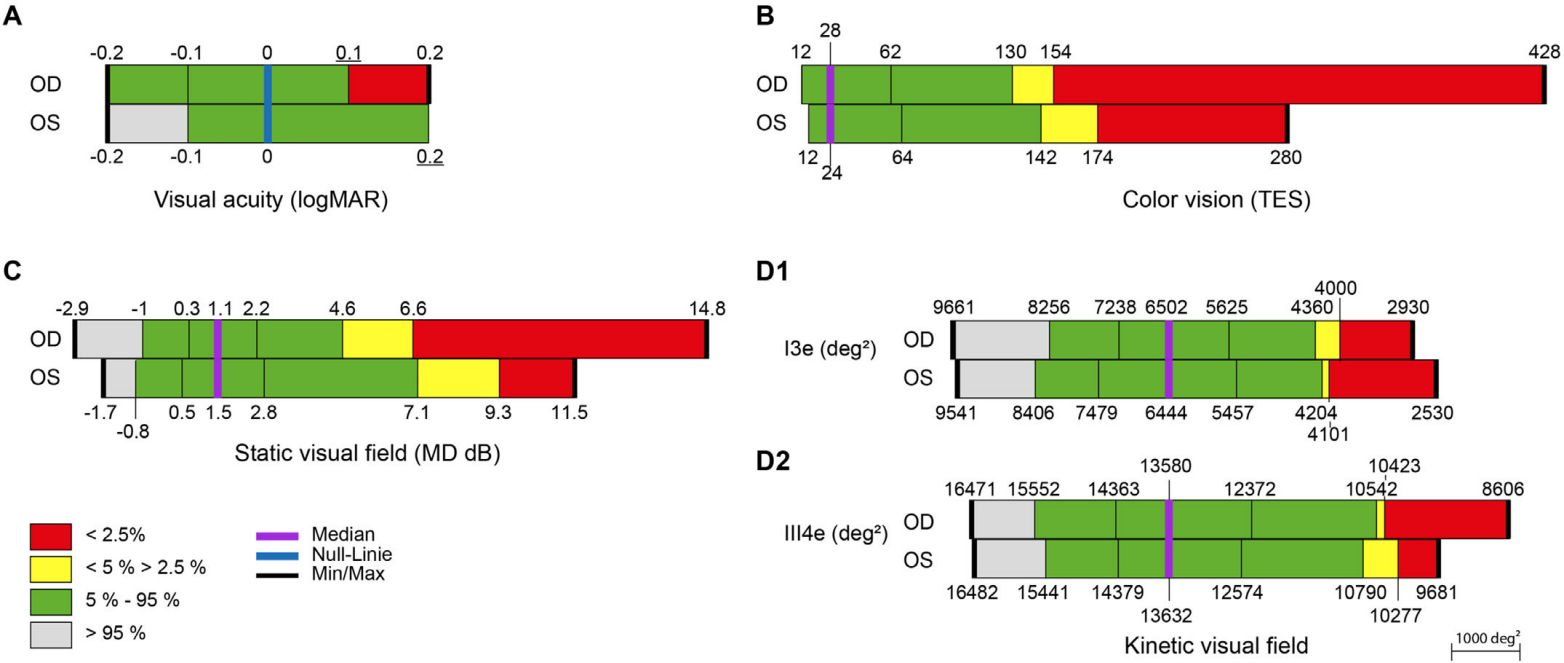
The distribution, for the 97 individuals, for the right (OD) and left (OS) eyes of BCVA (A, *top left*) Total Error Score of the Lanthony desaturated D15 test (B, *top right*), mean defect (C, *bottom left*), and I3e and III4e isopter area (D1 and D2), respectively (*bottom right*). *Gray shading* represents values that are better than the 95th percentile; *green*, values between the 95th and 5th percentiles; *yellow*, values between the 5th and 2.5th percentiles; and *red*, values that are worse than the 97.5th percentile. The two *black vertical lines* within the *green area* delineate the interquartile range. The median is represented by the *purple line* and the maximum and minimum values by the *bold black lines*. The scaling for isopter area (D1 and D2) is shown in the *bottom right*.

The descriptive statistics for the distributions in each eye at baseline of the BCVA, the TES, the areal extents of the III4e and I3e isopters, and the MD index for the 97 individuals are illustrated in Figure 4 and substratified by age group in Table 4.

**Table 4.** The Summary Statistics for the 97 Individuals, as a Whole, and Sub-Stratified by Age Group, for the Distributions in Each Eye of the BCVA, the Total Error Score of the Lanthony Desaturated D15 Test, the Mean Defect Index and the Areal Extents of the I3e and III4e Isopters.

Test	Parameter	Eye	Total (*n* = 97)	<60 y (*n* = 29)	60–69 y (*n* = 47)	≥70 y (*n* = 21)
BCVA (logMAR)	Mean ± SD	OD	−0.02 ± 0.08	−0.04 ± 0.07	−0.02 ± 0.07	0.00 ± 0.11
		OS	−0.01 ± 0.09	−0.03 ± 0.07	0.01 ± 0.09	0.00 ± 0.09
	Median [min, max]	OD	0.0 [−0.2, 0.2]	0.0 [−0.2, 0.1]	0.0 [−0.2, 0.1]	0.0 [−0.2, 0.2]
		OS	0.0 [−0.2, 0.2]	0.0 [−0.2, 0.1]	0.0 [−0.1, 0.2]	0.0 [−0.1, 0.2]
	Percentile [5%, 95%]	OD	[−0.20, 0.10]	[−0.20, 0.10]	[−0.10, 0.10]	[−0.20, 0.10]
		OS	[−0.10, 0.20]	[−0.10, 0.10]	[−0.10, 0.20]	[−0.10, 0.20]
Color vision (total error score, TES)	Mean ± SD	OD	44.39 ± 58.67	36.21 ± 53.24	42.00 ± 40.95	61.05 ± 91.08
		OS	44.08 ± 50.65	36.28 ± 50.01	43.62 ± 43.97	55.90 ± 64.26
	Median [min, max]	OD	28 [0, 428]	16 [0, 272]	28 [0, 154]	32 [0, 428]
		OS	24 [0, 280]	16[0, 184]	28[0, 142]	42 [0, 280]
	Percentile [5%, 95%]	OD	[0, 130]	[0, 130]	[0, 120]	[0, 126]
		OS	[0, 142]	[0, 174]	[0, 116]	[0, 118]
Static visual field, mean defect (MD dB)	Mean ± SD	OD	1.54 ± 2.22	0.82 ± 1.31	1.88 ± 1.88	1.77 ± 3.49
		OS	1.93 ± 2.40	1.50 ± 1.88	2.38 ± 2.84	1.50 ± 1.81
	Median [min, max]	OD	1.10 [−2.9, 14.8]	0.70 [−1.5, 3.1]	1.50 [−0.8, 7.9]	0.70 [−2.9, 14.8]
		OS	1.50 [−1.7, 11.5]	1.50 [−1.6, 5.4]	1.60 [−0.9, 11.5]	1.30 [−1.7, 6.3]
	Percentile [5%, 95%]	OD	[−1.0, 4.6]	[−1.5, 2.8]	[−0.4, 5.8]	[−1.0, 4.6]
		OS	[−0.8, 7.1]	[−1.6, 4.8]	[−0.6, 9.3]	[−0.8, 4.0]
Kinetic visual field I3e (deg²)	Mean ± SD	OD	6400.01 ± 1225.83	6919.79 ± 1205.04	6263.49 ± 1166.77	5987.76 ± 1197.78
		OS	6454.14 ± 1326.08	6911.24 ± 1266.88	6433.30 ± 1299.11	5869.57 ± 1284.27
	Median [min, max]	OD	6502 [2930, 9661]	6904 [4479, 9661]	6419 [3644, 8940]	5850 [2930, 7782]
		OS	6444 [2530, 9541]	6918 [4801, 9541]	6563 [2530, 8515]	5494 [4101, 8334]
	Percentile [5%, 95%]	OD	[4360, 8256]	[4937, 9116]	[4197, 7720]	[4371, 7672]
		OS	[4204, 8406]	[4877, 8657]	[4421, 8228]	[4179, 8181]
Kinetic visual field III4e (deg²)	Mean ± SD	OD	13,383.56 ± 1429.55	14,131.76 ± 1356.77	13,246.83 ± 1405.36	12,656.33 ± 1130.75
		OS	13,423.68 ± 1359.85	13,796.72 ± 1246.92	13,517.74 ± 1365.11	12,698.00 ± 1279.72
	Median [min, max]	OD	13,580 [8606, 16471]	14,363 [10542, 16471]	13,213 [8606, 16022]	12,547 [10402, 14298]
		OS	13,632 [9681, 16,482]	14,071 [11,414, 15,807]	13,641 [9681, 16,482]	12,694 [9909, 14,743]
	Percentile [5%, 95%]	OD	[10,542, 15,552]	[11,409, 16,161]	[10,501, 15,027]	[10,803, 14,261]
		OS	[10,790, 15,441]	[11613, 15619]	[11,613, 15,619]	[10,277, 14,306]

There was no obvious difference between the three age groups in either eye for the BCVA or the MD index. The TES and the areal extents of the I3e and III4e isopters each declined with increase in age for each eye. The summary statistics for the 97 individuals in each eye for the distributions at baseline of the BCVA, the TES of the Lanthony desaturated D15 test, the MD index, and the areal extents of the III4e and I3e isopters, for the group as a whole and by age, are given in Table 4.

The summary statistics for the distributions of the ERG, the BCVA, color vision, and perimetry (the grand mean [SD] of the mean of the two eyes of each individual) for the whole group and for the three centers at which more than 15 individuals were enrolled (centers 1, 2, and 3 in Annex 6) are given in Table 5 together with those of Coupland et al.^15^ and Birch et al.^16^ Table 5 also contains the summary statistics for the group, as a whole and by center, for the distributions of the TES for the Lanthony desaturated D15 test, the MD index, and the areal extents of the I3e and III4e isopters. The corresponding coefficients of variation (CVs) are given in Table 6. An evaluation of the CV for the BCVA (logMAR) was omitted due to the presence of division by zero values.

**Table 5.** Comparison of data from single sites with multicenter data, mean and SD

	Current Study					
Number of Individuals (n), type^a^ Age Mean (SD; Min–Max)	Total *n* = 97 (for ERG *n* = 95) Multi (11) 63.3 (7.9; 44–83) Mean ± SD	Site 1(*n* = 28) Single 63.8 (7.8; 44–79) Mean ± SD	Site 2(*n* = 18) Single 62.9 (5.9; 53–74) Mean ± SD	Site 3(*n* = 16) Single 64.9 (8.3; 53–83) Mean ± SD	Coupland et al.^15^ (*n* = 125) Single 39 (14; 19–72) Mean ± SD	Birch et al.^16^(*n* = 61) Multi (6) 68.7 (9.6; 39–90) Mean ± SD
DA 0.01 b-wave amplitude (µV)	220.7 ± 66.3	234.9 ± 68.7	202.8 ± 51.8	172.7 ± 28.1	282.6 ± 82.1	240.2 ± 109.4
DA 3.0 a-wave amplitude (µV)	−190.3 ± 6.8	−194.7 ± 54.9	−167.0 ± 39.5	−163.7 ± 29.8	−233.5 ± 69.2	−219.5 ± 68.7
DA 3.0 b-wave amplitude (µV)	316.9 ± 90.0	318.8 ± 86.6	299.6 ± 69.4	248.8 ± 39.4	387.1 ± 91.4	388.5 ±113.9
DA 12.0 a-wave amplitude (µV)	−228.1 ± 64.9	−227.3 ± 59.0	−210.2 ± 46.0	−188.2 ± 29.6		
DA 12.0 b-wave amplitude (µV)	327.7 ± 92.6	323.3 ± 85.8	318.0 ± 69.7	256.0 ± 35.8		
LA 3.0 a-wave amplitude (µV)	−29.8 ± 8.9	−33.2 ± 9.4	−27.4 ± 5.4	−22.3 ± 4.6	−38.5 ± 8.9	
LA 3.0 b-wave amplitude (µV)	119.8 ± 35.2	130.2 ± 28.4	111.4 ± 23.4	93.8 ± 21.7	143.2 ± 28.2	114.5 ±40.9
LA 3.0 30 Hz flicker amplitude (µV)	93.4 ± 25.8	104.6 ± 22.6	86.3 ± 22.3	74.7 ± 14.7	113.2 ± 24.0	110.9 ± 34.6
DA 0.01 b-wave peak time (ms)	97.6 ± 7.4	94.4 ± 6.6	99.5 ± 5.3	97.3 ± 8.4	90.0 ± 6.8	
DA 3.0 a-wave peak time (ms)	16.6 ± 0.8	16.4 ± 0.8	16.7 ± 0.6	16.3 ± 0.9	15.6 ± 2.2	
DA 3.0 b-wave peak time (ms)	53.5 ± 2.8	54.1 ± 3.5	52.8 ± 2.1	53.8 ± 2.8	48.2 ± 4.1	
DA 12.0 a-wave peak time (ms)	13.2 ± 1.1	13.1 ± 1.0	13.4 ± 0.9	12.4 ± 1.3		
DA 12.0 b-wave peak time (ms)	51.9 ± 4.0	52.6 ± 4.5	51.9 ± 3.8	50.1 ± 4.1		
LA 3.0 a-wave peak time (ms)	15.2 ± 0.7	15.2 ± 0.7	15.2 ± 0.5	14.7 ± 0.8	13.7 ± 1.2	
LA 3.0 b-wave peak time (ms)	30.9 ± 1.2	31.5 ± 1.3	30.1 ± 1.2	31.1 ± 1.1	30.5 ± 1.8	
LA 3.0 30-Hz flicker peak time (ms)	28.0 ± 1.5	28.8 ± 1.6	27.0 ± 1.3	28.1 ± 1.2	26.7 ± 2.4	
BCVA (logMAR)	0.0 ± 0.1	0.0 ± 0.0	0.0 ± 0.1	0.0 ± 0.1		
Color vision (TES)	44.2 ± 51.7	54.0 ± 37.4	43.7 ± 38.8	26.3 ± 27.5		
VF static (MD) db	1.7 ± 2.1	1.8 ± 2.0	1.7 ± 2.1	2.9 ± 2.9		
VF kinetic I3e (deg^2^)	6427.1 ± 1213	6399.0 ± 1180.1	6547.9 ± 903.0	5452.5 ± 1279.9		
VF kinetic III4e (deg^2^)	13,403.6 ± 1291.7	13,301.4 ± 1212.0	14,059.2 ± 1295.0	12,522.6 ± 1621.6		

Amplitude and Peak Time (Grand Mean ± SD of the Mean of the Two Eyes of Each Individual) for the ERG ISCEV Standard Responses (a-Waves, b-Waves, and Flicker Peak) under DA and LA Conditions, the TES for the Lanthony Desaturated D15 Test, the MD, and the Areal Extent of the I3e and III4e Isopters for the Whole Cohort and for Each of the Three Centers at Which More Than 15 Individuals Were Enrolled.

The values in the literature for the ERG, recorded using DTL electrodes, are given for comparison (Coupland et al.^15^ and Birch et al.^16^).

a type indicates Multicenter trial (``Multi'') or Single center (``Single'') followed by number of sites in brackets, if multicenter.

**Table 6.** The Coefficients of Variation (SD/Mean), Expressed as a Percentage, for the Data in Table 5

	Current Study					
Number of Individuals (*n*), type^a^ Age Mean (SD; Min–Max)	Total *n* = 97 (for ERG *n* = 95) Multi (11) 63.3 (7.9; 44–83) Coefficient of Variance	Site 1(*n* = 28) Single 63.8 (7.8; 44–79) Coefficient of Variance	Site 2(*n* = 18) Single 62.9 (5.9; 53–74) Coefficient of Variance	Site 3(*n* = 16) Single 64.9 (8.3; 53–83) Coefficient of Variance	Coupland et al.^15^ (*n* = 125) Single 39 (14; 19–72) Coefficient of Variance	Birch et al.^16^(*n* = 61) Multi (6) 68.7 (9.6; 39–90) Coefficient of Variance
DA 0.01 b-wave amplitude	30.1	29.2	25.6	16.3	29.1	45.5
DA 3.0 a-wave amplitude	29.9	28.2	23.7	18.2	29.6	31.4
DA 3.0 b-wave amplitude	28.4	27.2	23.2	15.8	23.6	29.3
DA 12.0 a-wave amplitude	28.5	25.9	21.9	15.7		
DA 12.0 b-wave amplitude	28.3	26.5	21.9	14.0		
LA 3.0 a-wave amplitude	30.0	28.2	19.8	20.5	23.1	
LA 3.0 b-wave amplitude	29.3	21.8	21.0	23.2	19.7	35.7
LA 3.0 30-Hz flicker amplitude	27.7	21.6	25.8	19.7	21.2	31.2
DA 0.01 b-wave peak time	7.6	7.0	5.3	8.7	7.6	
DA 3.0 a-wave peak time	4.7	4.9	3.5	5.4	14.1	
DA 3.0 b-wave peak time	5.2	6.5	4.0	5.3	8.5	
DA 12.0 a-wave peak time	8.6	7.9	6.3	10.7		
DA 12.0 b-wave peak time	7.7	8.5	7.3	8.1		
LA 3.0 a-wave peak time	4.8	4.8	3.4	5.7	8.8	
LA 3.0 b-wave peak time	4.0	4.1	3.9	3.4	5.9	
LA 3.0 30-Hz flicker peak time	5.3	5.5	4.8	4.3	9.0	
BCVA	N/A	N/A	N/A	N/A		
Color vision (TES)	116.8	69.2	88.7	104.7		
VF static (MD) db	118.8	113.8	120.8	100.0		
VF kinetic I3e (deg^2^)	18.9	18.4	13.8	23.5		
VF kinetic III4e (deg^2^)	9.6	9.1	9.2	12.9		

N/A, not done, because of division by zero values.

a type indicates Multicenter trial (``Multi'') or Single center trial (``Single'') followed by number of sites in brackets, if multicenter.

## Discussion

The study demonstrates, using identical recording devices, strict recording protocols, intensive training both of personnel and of potential participants, validation of centers, and continuous central ERG reading for quality control, that future multicenter trials of drug safety and efficacy can, in particular, use informative electroretinography and can also incorporate an extended age range of individuals. The latter finding is of importance given the increase in life expectancy and the potential for increasing age to affect the outcomes of clinical trials in ophthalmology. The data presented here can also serve as a basis for the calculation of the statistical power for future studies involving multiple centers (e.g., in treatment trials of rare retinal dystrophies, where a considerable number of centers are needed to achieve the required number of participants).

The ERG as an objective marker for changes in retinal function is a mainstay in testing certain drug products^17^ in the context of safety and efficacy;^18^ its importance is exemplified by the listing in ClinicalTrials.gov of 215 clinical trials (as of February 8, 2020, excluding the current study), using the search term *electroretinography*. Of these, 164 are ophthalmologic trials of which 110 use the Ganzfeld ERG, but only 24 are multicenter trials with more than 2 sites. Of these 24 multicenter trials, 14 involve more than 6 sites, and 4 of 14 involve more than 12 sites. The topics and the registration dates suggest an increase of the use of ERGs in multicenter trials involving rare eye diseases.

The current standard for the recording of the ERG is mandated by the International Society for Clinical Electrophysiology of Vision (ISCEV) with the aim of ensuring compatibility across centers.^19^^,^^20^ However, the standard does not consider many of the factors that can introduce differences in the outcome of the recording between centers.^21^ These factors include the stimulation and recording hardware; the type and positioning of electrodes^22^; the precise stimulus characteristics; the possible exposure to high levels of luminance, such as fundus fluorescein angiography, immediately prior to recording; the ability and training of the technicians; the accuracy of the peak identification, waveform cursor positioning, and waveform interpretation; and, in particular, artifact recognition.^23^ All such factors were addressed in the current study.

### Variances Associated with the ERG

The variances, expressed in terms of the CV, associated with the ERG peak times for the group as a whole and for each of the three centers that had enrolled more than 15 individuals (Table 6) were all less than 9% and all, with one exception, equal to or smaller (i.e., better) than the CVs of the only comparable single-center study in which DTL electrodes were used and the corresponding mean (SD) peak times were published.^15^

In the current study, the distributions of the ERG amplitude values were wider than those of the ERG peak times. This finding is compatible with that of the single-center study.^15^ The amplitude of the ERG is dependent upon the summation of all individual photoreceptor responses while the peak times are dependent upon the time course of the photoreceptor response, independent of the number of photoreceptors contributing to the response. Such differences are compatible with the reduction in the number and the alteration in morphology of the photoreceptors with age.^24^ Indeed, the mean ERG amplitudes decreased and the standard deviations increased with age, albeit slightly (Table 3); thereby, the CVs (not shown) become larger (i.e., worse) from the youngest to the oldest age groups. There was no such trend for the peak times.

The global CVs for the amplitudes were compatible with those from a similar multicenter study^16^ based upon individuals in whom the mean age was 5 years older than that of the current study (Table 6); the data were not available for the peak times. However, the global CVs for the amplitudes were slightly worse than the comparable single-center study.^15^ Such a finding was not unexpected given that the mean age of the individuals in the single-center study was approximately 25 years less than that of the current study. Nevertheless, site 3 in the current study returned consistently equivalent or better CVs than the other single centers; the reason for the better CV is unknown but could include greater homogeneity of factors such as dilated pupil sizes, age, axial length, and/or electrode positioning.

### Effects of Age on the ERG

The increase in peak time and the reduction in amplitude with increasing age for both the dark- and the light-adapted ERGs are compatible with that of previous studies.^25^^–^^28^ Rod and cone amplitudes exhibit an exponential decay with age.^27^ Similarly, the amplitudes in a septuagenarian age group are 25% to 40% lower than those between 20 and 50 years of age.^25^

The size of the natural pupil and also of that under mydriasis reduces with increasing age.^29^ The ISCEV standard (the 2008 update was applicable at the time of the study) stipulates that the pupil should be “maximally dilated” for the ERG. Three individuals in the present study had a maximum dilated pupil diameter of ≤4 mm, which attenuates retinal illuminance by approximately 0.5 log units compared to that for a pupil diameter of 7 mm. Given that small dilated pupils are not unexpected in a population of this age,^30^ the data from these individuals were included in the analysis.

### Variances Associated with BCVA, Color Vision, and Perimetry

Best-corrected visual acuity testing is highly standardized through the ETDRS procedures^31^ and is well established in clinical trials worldwide (ClinicalTrials.gov): two lines of letters (0.2 logMAR) or more can be reliably considered to represent clinical change.^32^ In the present study, the standard deviation associated with the BCVA was approximately 0.1 logMAR and was thus well within the two-line requirement.

The distributions of the TES, the MD index, and the III4e isopter area in the majority of individuals (5%-95%; green area in Fig. 4) were each wider in the left (second examined) eye, indicating that some individuals had adverse effects of test fatigue, which rendered a poorer outcome in the second eye examined. This is in agreement with the generally poorer performance of the TES^33^ and of standard automated perimetry for the second eye tested.^12^

The CVs for the baseline MD index in the current study were 124% in the right eye and 144% in the left eye, and both were better than that of 438% (*P* < 0.001) for the equivalent MD index, combined from two eligible baseline fields and taken across the two eyes in the Ocular Hypertension Treatment Study (OHTS).^34^ The differences in the CVs between the two studies are remarkable given the smaller standard deviation that would arise from the substantially greater number of individuals (1636) in the OHTS trial. Furthermore, contrary to the OHTS trial, the current study did not include an eligibility criterion for the MD index. The variability associated with the perimetric response increases in a nonlinear manner as the measured sensitivity declines (which, in itself, covaries with age) and is maximal at approximately 12 to 18 dB.^35^^–^^38^ Interestingly, only 6% of those in the OHTS trial manifested cardiovascular disease at baseline.^39^ It is also possible that the higher CV in the OHTS trial arose from the greater number of contributing centers compared to the current study.

A worsening in the MD index from a baseline of 3 dB is considered to represent a clinically significant event^40^ and has also been used as an endpoint in a safety trial of the antiepileptic drug vigabatrin.^41^ The range of the MD index within the current study indicates that a single criterion is an inappropriate approach. Such a finding is compatible with that of Cello et al.^3^ in benign intracranial hypertension, who proposed a worsening of the MD by 2 dB for those with a baseline MD of between 2 and 3.5 dB and of 3 dB for a baseline of between 3.5 and 7 dB. An alternative approach would have been to use an MD calculated from those stimulus locations exhibiting a sensitivity better than approximately 18 dB.^42^

The MD is an expression of the overall field loss and is adversely affected by disease of the primary visual pathway but also by cataract. The maximum MD in the current study was 14.8 dB compared to a theoretical value of zero. Outlying values of the MD are relatively common in an aged population and, in the current study, arose from the inclusion of several individuals with longstanding stable conditions. The alternative for summarizing the central visual field outcome (e.g., in the presence of cataract) would have been that of the loss variance (LV) index. This index is corrected for age, removes the loss expressed by the MD index, and is indicative of nonuniform (i.e., localized) loss. However, the use of the LV in the current study was considered inappropriate since it would have been insensitive to any possible generalized/uniform retinal dysfunction that was the emphasis of the study.

### Effects of Age on the BCVA, Color Vision, and Perimetry

The BCVA did not exhibit the previously reported age-related decline.^43^ The lack of such an outcome is likely to have arisen from the narrow age range of the majority of individuals, the improved acuity of those individuals with posterior chamber lens implantation following cataract extraction, and, possibly, the relative coarseness of the measurement (i.e., in logMAR decimal steps rather than in terms of the number of letters).

The age-related decline in the TES was higher for the oldest age group compared to the youngest age group and is compatible with that found previously.^33^ The decline can be attributed to changes in the crystalline lens and/or to age-related changes at the fovea.

The MD index, as would be expected, did not vary with age in either eye since the index represents the mean, across all locations within the central field, of the difference between the age-corrected normal value and the corresponding measured value.

The study used Program G1, which has more paracentral stimulus locations compared to the conventional Humphrey Field Analyzer that uses the Central 24-2 or 30-2 threshold tests or even the newly introduced Central 24-C threshold test. Program G1 is, therefore, more sensitive in detecting potential foveal and parafoveal abnormalities occurring in age-related retinal changes.

The age-related decline in the areal extent of the reaction time–corrected I3e and III4e isopters is consistent with that reported for both manual and semiautomated kinetic perimetry.^11^^,^^44^ The isopter measured by semiautomated kinetic perimetry is referenced to the corresponding age- and reaction time–corrected normal isopter. However, the difference is not expressed in terms of a single index. Such an index, comparable to that of the MD, would be clinically beneficial. The volume of the visual field may serve as such a parameter in future studies.^45^^,^^46^

The cohort included some individuals with moderate age-related cataract and/or other ophthalmologic conditions common in advanced age to the extent that they were not considered an exclusion criterion. The presence of moderate age-related cataract does not affect either the peak time or the amplitude of the Ganzfeld ERG^47^; however, such manifestation does adversely influence the visual field measurement.^48^^–^^51^ Sensitivity to smaller perimetric stimuli is attenuated by age and by cataract to a greater extent than that to larger stimuli.^48^ In the current study, the proportionate reduction between the youngest and old groups in the median areal extent across the two eyes was 18% for the I3e isopter and 11% for the III4e isopter.

## Conclusion

The data presented here from an international multicenter study illustrate the benefit of identical equipment, stringent on-site instruction and training, and, for ERG, the quality control and timely feedback to investigators by a centralized procedure involving highly experienced central readers providing consistency of interpretation. The data sets, together with the study protocols for instruction and training, for quality control and validation, and for measurement acquisition, may be useful in planning ophthalmologic multicenter studies in which electrophysiologic and visual function testing are used to monitor safety and/or efficacy.

## Supplementary Material

Supplement 1
