# Supplementary material for: Quality Control Procedures and Baseline Values for Electroretinography, Perimetry, Color Vision, and Visual Acuity in an International Multicenter Study: Observations from a Safety Trial in Chronic Stable Angina Pectoris
Source: Transl Vis Sci Technol. 2020 Jul 28;9(8):38. doi: 10.1167/tvst.9.8.38 (PMC7422805; doi:10.1167/tvst.9.8.38)
Supplement: Supplement 1 [file tvst-9-8-38_s001.pdf]

Annex 1

Visual Field, centre validation

Before the start of the study (i.e. first patient inclusion by the corresponding “main investigator) each selected centre in charge of performing perimetry will be validated by the the specialists in perimetry of the Scientific Ophthalmic Safety Committee.

1. Main objectives of the validation process:

The main objectives of the validation process are to validate each centre on the quality / reliability of the recordings and to confirm the feasibility of the electronic transmission from the investigator’s centre to the central data base .

2. Performance of the quality review:

The quality assessment of the visual field examinations will require the following steps:

2.1. At the investigator’s site:

The ophthalmological investigators will :

- select / enrol volunteers (no age range pre defined) after having checked that they have not participated in repeated visual field studies during the three years preceding this procedure ; therefore, the quality of the instruction given by the centre to the volunteer being investigated will be assessed , in addition any potential learning effect will be avoided.
- perform the VF measurements (static and kinetic) in three selected healthy volunteers according to the procedure described in the study protocol (see supplement 3)
- transmit electronically to the central data base the visual fields (graphs / plots/ calculated values) of 3 healthy volunteers (the visual fields from the 3 should be sent together) .

2.2. Quality Control (QC) validation procedure of perimetric examination

- The visual electronic version of the visual field records will be reviewed and assessed by the independent quality control board of specialists in perimetry, members of the Scientific Ophthalmic Safety Committee.
- The assessment for validation will be done on centers identification being coded (blind assessment of the center) within a maximum of 15 days upon receipt (at the central data base).

2.2.1 Quality criteria of semi-automated kinetic perimetry

1) The appropriate program and stimuli should be used (according to the protocol requirement see supplement 3).
2) The blind spot should be represented as an absolute scotoma. with the I4e stimulus,
3) **The blind spot size should be smaller than 5 degree**2,
4) **The individual patient Reaction time should be between 200 and 1500 ms,**
5) The isopters should consist of continuous circular lines,
6) The isopters should not cross each other,
7) The area of the visual field should be within the age corrected normal range,
8) All appropriate information should have been entered into the computer and should appear on the visual print-out.

2.2.2. Quality criteria of automated static grid perimetry (thresholding strategy)

1) The appropriate program and stimuli should have been used
2) The Mean defect value (MD) of the static perimetry should be within the corrected normal range
3) **The incorrect responses to the false-positive, false-negative or fixation control catch trials should each not exceed 30% in either eye.**

3. Certification of centres

The certification of each centre will issued and signed by the specialists in perimetry after having validated that each centre fulfils the above mentioned criteria

-A validation certificate will be issued and automatically transmitted to central data base, the investigating ophthalmologists and the Sponsor

- The central data base manager will be responsible for the transmission of the information to the CCTRA (Centre for Computerised Treatment Allocation).

No patient can be pre-selected (ASS0 visit) for the study by any center (i.e. the main investigator's) prior to the issue of this validation certificate.

Annex 2

Colour Vision Testing: Panel D-15

Protocol:

Colour vision will be evaluated with the desaturated Panel D-15 test.
The test will be performed for each eye without pupil dilatation, on near vision with optimal optical correction, and during constant cold lighting (D65, 270 lux is recommended). The subject will be instructed to classify 15 numbered discs of different desaturated chromaticities. To avoid errors dues to incomprehension of the test or lack of attention, the examination can be repeated.

Results:

The investigator will link himself to the website (www.belatorok.com/d-15) and enter the numbered caps according to the order done by the patient.

The Total error score (TES) is automatically calculated according to Lanthony's method (1986, quoted at the web site). The evaluation is performed by a computerized analyzer and is automatically and immediately sent back electronically to the investigator. The investigator will make a print-out of this document which will be considered as source data and will be added to the patient's file.

Annex 3

Visual field assessment with the Octopus 101 perimeter

Static and kinetic perimetry will be undertaken on each eye using the Octopus 101 perimeter, a standard commercially available instrument used in ophthalmology.

Static threshold perimetry will be undertaken within the central field (i.e. within a radius of 30° from the fixation point). The threshold response (i.e. the minimum brightness of a spot of light [the stimulus] necessary to evoke a response within the visual system) will be determined using Goldmann stimulus size III (subtending 0.431° at the eye) at each of 64 locations within the central field. The subject is required to indicate, by pressing a response button, whenever he/she perceives the spot of light.

Semi-automated kinetic perimetry (SKP) of the peripheral visual field (i.e. the visual field beyond an eccentricity of 30°) will be undertaken by separately presenting two moving spots of bright white light from the periphery towards the centre (the stimulus vector) at 15° angular intervals (i.e. meridian) at an angular velocity of 3°/s. The size of the two spots of light will be Goldmann III and Goldmann I (subtending an angle of 0.108°at the eye) and the brightness 1000asb and 100asb, respectively. The subject is again required to indicate, by pressing a response button, whenever he/she perceives the spot of light. The line joining the location along each meridian at which the given stimulus is detected is termed the isopter.

The spots of light for both static and kinetic perimetry are presented against a white background of 10 cd/m^2.

Technical operation

The patient will be instructed in the required procedures and will undergo a short practice session prior to the start of the formal visual field examination. The distance refraction, corrected for a distance of 40 cm (i.e. the radius of the cupola of the Octopus 101 perimeter) will be worn in the form of thin-rimmed lenses positioned close to the eye in order to avoid shading parts of the visual field. The patient's lashes should not touch the lens. Each eye will be tested separately by covering the fellow eye with an opaque occluder. The subject´s pupils should not have been dilated prior to the visual field examination. The patient is positioned with the forehead touching the head rest and the chin in firm contact with the chin rest in such a way that the eye under examination is gazing at the fixation target (four green bars forming a fixation cross around the geometrical centre of the cupola). The noise level within, and surrounding, the examination room should be sufficiently quiet to enable the subject to concentrate on the task. The examination should ideally be undertaken in darkness although some minimal local ambient illumination is acceptable. Any change in the general lightening conditions within the room or the introduction of other distracters during the examination (e.g the telephone ringing, etc) should be avoided. The subject holds a response button in his/her hand which is pressed to indicate whenever he/she sees the (static or kinetic) stimulus. It is also necessary to ensure that the subject’s sitting position is comfortable and relaxed, and that the chair is adjusted accordingly, so that the subject can easily tolerate the examination. Additionally, a small camera within the perimeter permanently records the position and

movement of the subject's pupil. The technician has to ensure that the head is adjusted so that the pupil is aligned in the centre of the monitor.

The instructions to the patient for static perimetry of the central field will be modified from those used for the Ocular Hypertension Treatment Study [OHTS]), namely:
"Always look straight ahead at the centre between the four green bars (the fixation target). Other lights will flash one at a time at other positions around the centre of the bowl. Some lights will be bright, and others will be dim. Press the button whenever you see one of these flashes. You are not expected to see all of them. The best time to blink is just as you press the button."

The instructions for static perimetry will be adapted for kinetic perimetry of the peripheral visual field. The patient will be reminded again to look steadily at the fixation target and press the response button whenever they become aware of a light moving into the edges (the periphery) of their vision.

Preparatory test procedure

Step 1

Kinetic perimetry will be performed with stimulus III4e, presented at an angular velocity of 3°/s along each of 16 meridians of the visual field (i.e. at 15° intervals). The order of the meridian examined is selected randomly by placing the origin of the vectors directed from periphery towards the centre, 10° outside of the age-corrected normal reference isopter. The direction of the vectors should be chosen in a way that the presumed visual field border/isopter is crossed by the vector almost perpendicularly. If there is a suspicion of an abnormal visual field (e.g. diabetic retinopathy or hemifield defect, e.g. after stroke) the patient may not see the vector movement in those regions affected by the disease. In this case, the technician will move the vector further in towards the centre of the field until the subject recognizes the stimulus. For quality assessment reasons, reaction time is assessed separately; for this purpose three "reaction time vectors" are placed within the central 30° radius of the intact visual field. The software will automatically correct the isopters for the individually measured reaction time.

Step 2

The smaller and less bright I3e stimulus will be presented at an angular velocity of 3°/s in the same manner as that described for the III4e stimulus, described above.

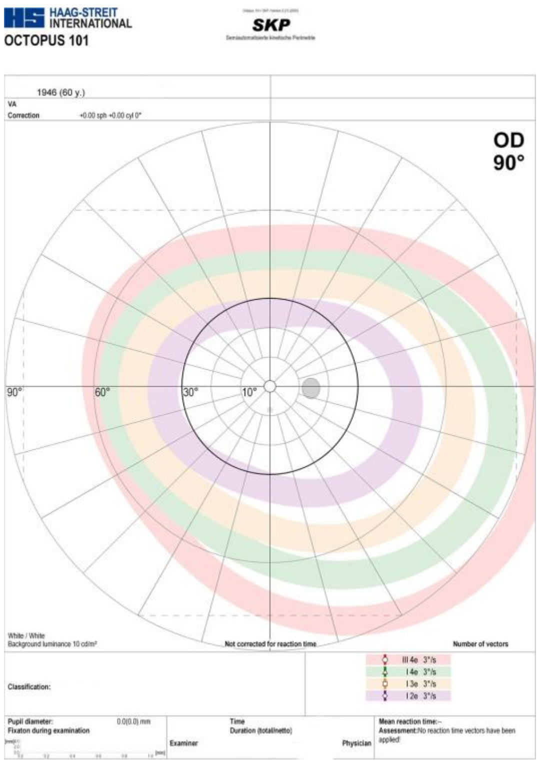

Figure 1 *The reference intervals for the normal III4e (pink) and I3e (orange) isopters recorded with the Octopus 101 perimeter*

Step 3: Blind spot assessment

At least 8 vectors (i.e. 30° apart and randomly selected) originating from the assumed centre of the blind spot and moving at an angular velocity of 2°/s are presented using the I4e stimulus.
The area of the blind spot is automatically calculated and the areal measure serves as a quality criterion of the patient’s cooperation and attention. Magnification software is used to assess the blind spot more accurately.

Step 4, 5, and 6: The same procedure will be applied to the fellow eye after a rest period of 5 minutes.

Subsequently the area encircled by each isopter will be calculated.

Step 7: Automated static threshold perimetry

After a 5-10 min. rest period, automated static perimetry will be undertaken. The subjects will be instructed in the new task, namely that the spot may appear in different locations with different brightness levels and that he/she has to press the button each time he/she perceives the spot, even if it is very dim, and that not all (only approximately 50% of the) presentations are expected to be perceived. A modified Program G1/G2 grid (incorporating one additional central test location, one test location within the centre of the blind spot and four additional test locations within the immediate vicinity of the blind spot) will be used, as show below:
Altogether 64 locations are examined within the 30° central field, as illustrated in the table.

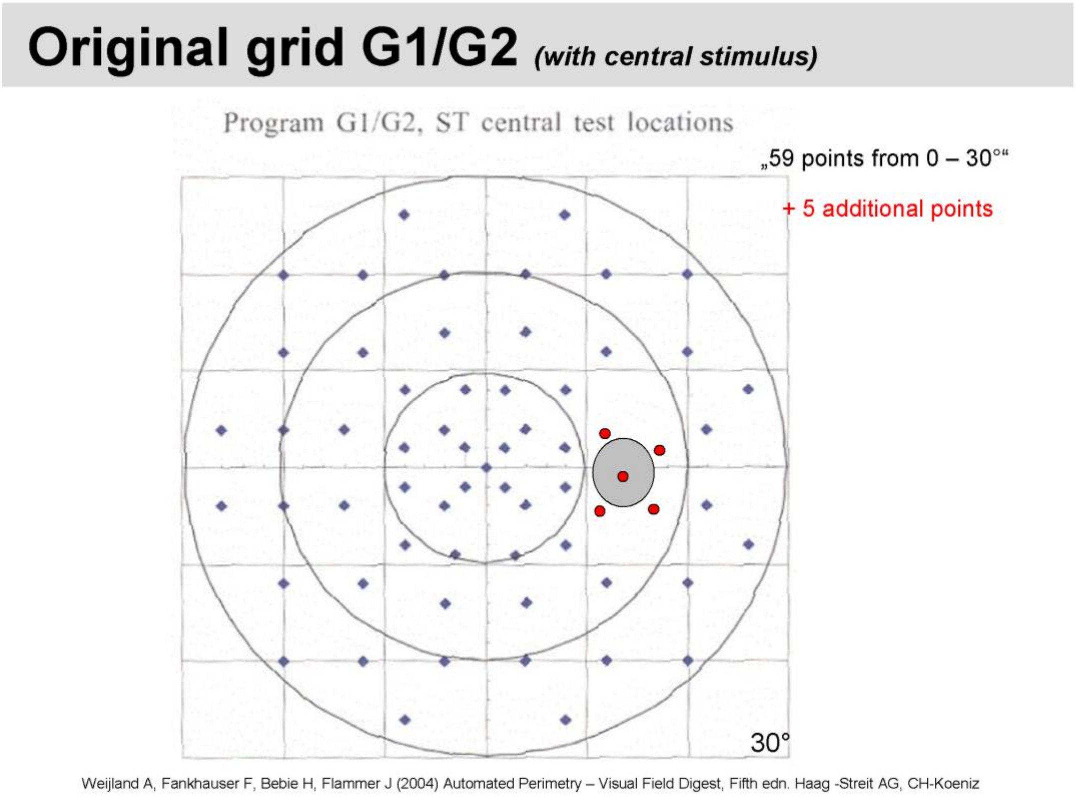

Figure 2 illustrating the spatial configuration of the 64 test locations.

Nr.	x	y	angle	ecc
1	4	4	45	5.65685425
2	-4	4	135	5.65685425
3	-4	-4	225	5.65685425
4	4	-4	-45	5.65685425
5	8	8	45	11.3137085
6	-8	8	135	11.3137085
7	-8	-8	225	11.3137085
8	8	-8	-45	11.3137085
9	4	14	74.0546041	14.5602198
10	-4	14	105.945396	14.5602198
11	-4	-14	254.054604	14.5602198
12	4	-14	-74.054604	14.5602198
13	-20	4	168.690068	20.3960781
14	-20	-4	191.309932	20.3960781
15	26	8	17.102729	27.202941
16	26	-8	-17.102729	27.202941

17	2	2	45	2.82842712
18	-2	2	135	2.82842712
19	-2	-2	225	2.82842712
20	2	-2	-45	2.82842712
21	4	20	78.6900675	20.3960781
22	-4	20	101.309932	20.3960781
23	-4	-20	258.690068	20.3960781
24	4	-20	-78.690068	20.3960781
25	-14	4	164.054604	14.5602198
26	-14	-4	195.945396	14.5602198
27	22	4	10.3048465	22.3606798
28	22	-4	-10.304846	22.3606798
29	-26	4	171.253838	26.3058929
30	-26	-4	188.746162	26.3058929
31	20	20	45	28.2842712
32	20	-20	-45	28.2842712
33	0	0	0	0
34	8	2	14.0362435	8.24621125
35	-8	2	165.963757	8.24621125
36	-8	-2	194.036243	8.24621125
37	8	-2	-14.036243	8.24621125
38	12	20	59.0362435	23.3238076
39	-12	20	120.963757	23.3238076
40	-12	-20	239.036243	23.3238076
41	12	-20	-59.036243	23.3238076
42	20	12	30.9637565	23.3238076
43	-20	12	149.036243	23.3238076
44	-20	-12	210.963757	23.3238076
45	20	-12	-30.963757	23.3238076
46	2	8	75.9637565	8.24621125
47	-2	8	104.036243	8.24621125
48	-3	-9	251.565051	9.48683298
49	3	-9	-71.565051	9.48683298
50	12	12	45	16.9705627
51	-12	12	135	16.9705627
52	-12	-12	225	16.9705627
53	12	-12	-45	16.9705627

54	-20	20	135	28.2842712
55	-20	-20	225	28.2842712
56	8	26	72.897271	27.202941
57	-8	26	107.102729	27.202941
58	-8	-26	252.897271	27.202941
59	8	-26	-72.897271	27.202941
60	12	3	14.0362435	12.3693169
61	14	-1	-4.0856168	14.0356688
62	17	1	3.36646066	17.0293864
63	17	-5	-16.38954	17.7200451
64	12	-5	-22.619865	13

Table denoting the 64 test locations.

At the initial visit, static perimetry will be undertaken using a 4-2-1(1) dB staircase threshold assessment strategy: the initial stimulus is presented at a luminance level of 4 dB above or below the assumed threshold at the given location. The stimulus luminance is then increased / decreased in 4 dB steps until the stimulus is no longer perceived / perceived for the first time (→ reversal). Thereafter, the procedure is repeated in 2 dB steps until the next reversal is achieved and repeated again until a final reversal with a 1 dB step size is achieved.
For each subsequent visit, the local threshold value obtained at the previous examination for the given patient is used as the local initial value for the starting point of the staircase in order to reduce the examination duration.

The local differential luminance sensitivity is estimated using a maximum likelihood (ML) method.

The thresholding procedure is carried out for each of the 64 locations in random order. It should be noted that the three staircase/bracketing procedures are not undertaken for each location in a single sequence. Instead, the computer stores the values of a given reversal at a given location. Specifically, it records the last value of the stimulus and the patient's response and selects the next location in random order. This is done in order to avoid local adaptation and loss of attention in regard to any given single test location. Thereby, each threshold sequence is put together by multiple events at each location with these events being presented in the correct order of brightness steps at each different location and at different time points. If the patient encounters a problem in response at any location, the thresholding sequence can be re-checked for the particular location(s).

Subsequently, the results are stored in an electronic database and also printed out on paper. The results include the number of incorrect reponses to the false-negative and to the false-positive catch trials, the magnitude of the mean deviation (MD) and other visual field indices as well as a display of the continuous video-pupillographic fixation control. Additionally, the printout contains visual acuity, refractive correction used, the level of the cupola luminance, a display of all vectors utilised for SKP, the mean reaction time for SKP, and the area of each of the two isopters and of the blind spot, corrected for the individual reaction time of the patient.

Calibration

The perimeter has an inbuilt self-calibration procedure that is activated automatically when the instrument is switched on. This procedure checks the luminance of the test and background stimuli. An error message is displayed if the calibration fails.

All examination data are stored together with the patient ID data on a computer file, printed out and, in parallel, electronically submitted to the central server.

Annex 4

ERG study protocol

Standard Ganzfeld electroretinogram (ERG)

Check all patient details and record appropriate data. Explain the procedures in full, and answer any questions the patient may have. Fundus photography should be avoided to be done within the hour preceding the start of the ERG procedure. Explain the effects and consequences of the eye-drops that are being used.
The ERG will be recorded according to the ISCEV Standard for Full-field Clinical Electroretinography (see https://iscev.wildapricot.org/standards).

The following document serves to specify those parts where the standard recommendation (in the present paper 2004 update) allows several alternatives and to describe the exact procedure and stimulus parameters to be followed by any investigating centre.

The patient should have dilated pupils for all ERG recordings. Tropicamide is preferably recommended to be used .
The electrodes are best fitted immediately after the installation of the dilating drops and prior to dark adaptation. DTL-Fibre electrodes are used for recording corneal responses. The use of topical anaesthesia is not necessary in case of the use of fibre electrodes. The reference electrodes should be placed on the zygomatic fossae. The ground electrode is placed on the central forehead. Impedances should be measured and be approximately the same for all three electrodes and $\leq 5k\Omega$.

The patient should then be positioned on the Ganzfeld chin rest, and the height of the chin rest, stool and table adjusted for maximum patient comfort and to align the subject's eyes with the central fixation spot and monitoring camera if fitted. DTL-Fibres electrodes will be used and provided to the centres by the Sponsor to ensure the homogeneity among the centres and the reliability among centres. The position of the transcorneal fibre in relation to the pupil should be recorded. The investigator must ensure that the fibre electrodes are always at or just above the rim of the lower lid. Pupils size should be recorded at the end of the session with a comparison ruler.

Dark adapted (DA) ERG:
Dark adapt the patient for a full 20 minutes. This is best achieved in the test room and should be in total darkness. All light within the patient's section of the room should be extinguished, including any red 'safe' light. The patient should be attended at all times, preferably by the recording technician as well as any accompanying person. Curtains should be used to screen the subject from the instrument lights and screens. If it is unavoidable that the patient and operator cannot be screened off from each other, screen illumination should be reduced to the minimum required for operating comfortably once the operator has dark adapted. If it is necessary for the operator to leave the room during dark adaptation, then the patient should be asked to close their eyes whilst this happens, and also whilst the operator re-enters the room. Preferably a black curtain should shied the door. The outside illumination should also be reduced to allow minimum light contamination during re-entry.

Fit the fibre electrodes under the lowest possible level of red illumination, (use the minimum working level for the red light that is acceptable to the operator, best achieved where the operator has dark adapted with the patient), check and adjust if necessary the position of the fibres relative to the pupils under red light. Record whether change of electrode position was required. Position the patient on the chin rest in the previously adjusted Ganzfeld bowl. The patient should be instructed to look towards the fixation lamp, which is used on its lowest intensity setting. Patient fixation is monitored, if possible, using the infra red (IR) camera system within the bowl. Dark adapt the patient for a further 5 minutes.

The recordings detailed below should be recorded in all patients. Any variation in protocol, and the reasons for doing so, must be documented in the patients file. Dark adapted recordings should be obtained first with the lowest stimulus intensity, then successively with increasing intensity. Each intensity level should be replicated and recorded twice, and if good reproducibility is not present, further repeat(s) should be performed. Allow an inter stimulus interval (ISI) of 5 seconds between flashes at the low-intensity; of 10 seconds at the intermediate and higher intensities, and of 20 seconds at the upper levels of intensity. When the DA ERG responses have been satisfactorily recorded, the patient should be restored to photopic conditions using the background lighting of the Ganzfeld set to approx 34 cd/m_2.

Light adapted (LA) ERG:
The patient should be reminded to keep his eyes fully open during light adaptation. The LA photopic recordings should be performed after 10 minutes light adaptation, again with the necessary number of replications. Each averaged response should be recorded twice. The background light should remain on at all times during photopic recording. When recording is complete, measure and record pupil diameter. Remove all electrodes and remind the patient of the effects of mydriasis routine safety precautions measures should be carefully followed.

Notes on recording the ERG

It should not be necessary to average the DA ERG data, but this may be needed if the waveform is poor or contaminated by artefact. Average enough to “clean up” the waveform form ERG, from artefacts but remember to maintain the allowed inter-stimulus intervals (ISI) at all times. Prior to every flash, instruct the patient to fixate, sit still and try not to blink or move their mouth until after the light has flashed, and open their eyes (comfortably) wide. Monitor patient compliance on the infrared camera display if possible. The patient should be told to keep their jaw and face relaxed, and to avoid clenching their teeth. Their eyes should always be open and looking at the fixation light in the Ganzfeld bowl.

Averaging is usually beneficial for good light adapted recordings. Start the 30Hz flicker stimulus and allow to run for 1-2 seconds before commencing averaging. 10-30 averages per trial should be adequate if the patient can maintain eye opening without excessive EMG (electromyographic, muscle artefact). For the “single flash” photopic ERG (“LA 3.0”), again start the flashing prior to commencing averaging. Flash at 1Hz; 5-10 averages per trial should be ample. Use a time-base of 200ms for DA ERGs and 100ms for LA ERGs. Filters should be 0.3 – 1000.
Any technical difficulties, such as an inability to maintain full pupil exposure during photopic flicker ERG recording should be recorded.

Create back-up of ERG data on CD at the end of each recording session.

Required stimulus parameters

The white standard flash (SF) should be set at 3 $cd.s/m^2$ with 4 ms stimulus.

a) Dark adapted ERG: For each response the stimulus intensity will be as follows:

- DA 0.01 (Rod ERG):	SF-2.5 log units=0.009 $cd.s/m^2$ (minimum ISI 5 seconds);
- DA 3.0 (rod/cone ERG)	SF 3.0 cd.s/m2 (Standard ISCEV flash) (minimum ISI 20 seconds);
- DA 12.0 High intensity rod/cone ERG:	SF +0.6 log units=12 cd.s/m2 (minimum ISI 20 seconds).

b) Light adapted ERG: After 10 minutes of light adaptation at 34 cd/m^2:

LA 3.0 (30 Hz cone ERG):	SF 3.0 $cd.s/m^2$;
- LA 3.0 (Single flash cone ERG) :	SF 3.0 $cd.s/m^2$ with an ISI of 0.5 seconds.

Annex 5:

ERG centralised procedure
(Qualitative and quantitative review)

I- Introduction

Objective of the centralised procedure:

The objectives of centralising the ERG are to:

- First validate each centre prior to the inclusion of their first patient,
- Second, to:
  - ensure the quality of the recordings throughout the entire study,
  - inform the investigators of the eventual observed technical errors, inaccuracy of the measurements, moreover on the quality of the recording, etc…,
  - to ensure a uniform and standardised evaluation of all data from all centres. This is possible because of the use of identical equipment (Espion E2, DTL electrodes) in all sites and a centralised procedure.

II- ERG REVIEW PRIOR TO THE START OF THE STUDY (i.e. validation procedure)

Before the start of the study, three ERG tests will be performed by each centre in order to validate each centre on the quality of the recordings and on the feasibility of ERG procedure including the electronic transmission to the central data base. The three ERGs should be sent together.

The data will be fully anonymised and the evaluation will be performed blind treatment, patient identification and to the site.

The quality validation will be done by the central reviewer within a maximum of 15 days upon receipt. A copy of the validation certificate will be transmitted to each investigator, to the main investigator of the corresponding centre, and to the sponsor.

The centre cannot start to include patients until the centre has not been approved by the central reader. Therefore, it is only after validation by the central reader that a centre will be formally admitted to the study and become able to take data from patients.

III- ERG PERFORMANCE BY THE INVESTIGATORS DURING THE STUDY

ERG will be performed by the investigator according to the “ERG procedure” described in Supplement 1.

ERG will be digitally recorded using the ESPION E2 equipment and DTL - fibre electrodes.

Before the start of an ERG recording, a “patient’s file” will be created by the qualified technician(s) pre-selected in the centre. This file will contain the patient ID number, his/her date of birth (d.o.b.), and gender (these information are mandatory as the clinical assessment is done in comparison with the expected values of an age matched population).

The ERG will be performed by a qualified technician. The amplitude (μv) and peak time (ms) of each component (a- and b-waves, flicker response) will be measured by cursor positioning. The cursor positioning will be marked on the waveforms. Prior to the start of the recording, the technician will ask the patient his/her date of his/her birthday in order to check that the reported personal data are correct.
At the end of the ERG recording procedure, the technician will hand over to the investigator (OPH/electrophysiologist) the waveforms for review/validation prior to the sending to the central data base (the technician does not have the “right” to send the waveforms to the central data base). The investigator will review the ERG performed by the technician, eventually will reposition the cursor(s) (i.e. re-measurement) and make the clinical assessment of the ERG. The so called “reviewed ERG” will be printed-out (source document to be included into the patient’s medical file) and the completed electronic document of the ERG will be saved on the Espion E2, then transmitted to the central data base. In addition a back-up (CD) of all electronic waveforms will be performed.

The ERG tests should be sent using an electronic transmission to the Central Data Base immediately after their performance (i.e. within the day of recording). Within 5 days upon receipt at the central reader’s office the qualitative and quantitative assessments of the central reader will be received by the ophthalmologist (investigator).

IV- ERG CENTRAL REVIEW DURING THE STUDY

IV-1: ERG sequences description

The central reviewer will have a direct access to the central data base up loaded with the ERGs performed/validated by the investigators.

For each ERG recording (i.e. dark/light adapted) a qualitative and quantitative analysis will be performed by the central reader (measurement of amplitude and implicit times of each component). This review has to be done within 5 days upon receipt.

The baseline ERG must be of high quality as all other waveforms will be compared to this reference one (so called baseline). Therefore, if the central reviewer is:
- satisfied with the quality of the ERG performed at the selection visit (ASS1), this one will become “the baseline ERG”. This “validation” will be transmitted to the computerised centre for randomisation and treatment allocation (CCRTA).
- not satisfied with the quality of the ERG performed at the selection visit, an additional ERG will be requested by the central reviewer to be done by the investigator (OPH) at the pre-inclusion visit within a maximum of 7 days. Then the same quality process will take place:

  - if the quality is still insufficient the patient will not be eligible,
  - if the quality is good, this ERG performed at the pre-inclusion visitwill be the baseline ERG; its validation will be transmitted to the CCRTA and the patient will be potentially eligible as regards to this criteria.

IV-2: ERG central review procedure

The central reader will assess the ERG quality and will be responsible for its analysis and measurements. This will be done for any ERG recorded (i.e. all visits performed).

The quality and quantitative evaluation of the central reader will be sent to the investigator within 5 days working upon receipt.

An overall medical review will then be done for each completed patient.

IV-2-1: Quality control

The quality control will be done on:

- the signal-to-noise ratio,
- the repeatability of identical stimulus and recording conditions,
- the number of artefacts that occur at critical phases in the ERG (e.g. near the normal b-wave maximum, etc.),
- on the basis of the values being within the age-matched normal range in terms of amplitude and implicit times,
- the proper setting of the cursors for baseline a-wave minimum, b-wave maximum,
- the proper recording of important parameters (pupil size, impedance values, light calibration values, timelines such as time of light and dark adaptation),
- the completeness of the recordings concerning each stimulus parameter of the protocol,
- the promptness of the response of the centre to request from the central reader.

IV-2-2: ERG measurements

The central reader will assess the investigator's reported measured components of the different responses and may or may not re-position the cursor(s) according to his evaluation. All data after having been assessed will be either validated (measured value agreed) or modified (measured value not agreed upon i.e. a re-positioning of the cursor being done). These values will be entered directly into the "Central Reader Database". The main analysis of the ERGs will be performed with the data from the central data base.

The following responses to light stimuli will be reviewed/measured:

Dark adapted phase

- rod b-wave amplitude (μV) and peak time (ms),
- standard combined rod/cone ERG amplitude (μV) and peak time (ms),
- high intensity rod/cone ERG amplitude (μV) and peak time (ms)

Light adapted phase

- flicker response amplitude (μV) and peak time (ms),
- Single flash a- and b- wave amplitudes and peak time

ANNEX 6

ERG of three sites of the study with more than 15 individuals

Abbreviations: **OD/OS:** oculus dexter/oculus sinister; **DA**: dark adapted; **LA** light adapted **AMPL:** Amplitude (μV); **PT:** peak time (ms); **MD:** mean deviation (db); **TES:** total error score
CV: color vision; **VFs:** visual field static (center); **VFk:** visual field kinetic (periphery); **BCVA:** Best corrected visual acuity; **CoV:** Coefficient of Variance (%); **SD:** Standard Deviation

ERG Rod response, DA 0.01 b-wave

test	parameter	OD/OS	parameter	Site 1 Argentina	Site 2 Hungary	Site 3 Portugal
				N=28	N=18	N=16
				Age years Mean+/- SD 63.8 ± 7.8	Age years Mean+/- SD 62.9 ± 5.9	Age years Mean+/- SD 64.9 ± 8.3
DA 0.01 b -wave	AMPL (μV)	OD	Mean+/- SD	231.4 ± 68.8	204.4 ± 51.7	172.7 ± 30.4
			Median [min, max]	240.6 118.4 ; 393.6	194.2 129.1 ; 298.5	165.3 131.0 ; 235.4
			CoV %	29.7	25.3	17.6
			Percentile [5%, 95%]	[122.9 ; 324.6]	[129. 1 ; 298.5]	[131.0 ; 235.4]
			Quartiles [25%, 75%]	[175.6 ; 283.9]	[163.7 ; 249.3]	[147.3 ; 193.2]
		OS	Mean+/- SD	238.3 ± 74.2	201.1 ± 57.4	172.7 ± 28.9
			Median [min, max]	217.5 133.7 ; 407.2	192.6 120.8 ; 342.9	168.3 125.6 ; 221.0
			CoV %	31.1	28.5	16.8
			Percentile [5%, 95%]	[138.9 ; 374.6]	[120.8 ; 342.9]	[125.6 ; 221.0]
			Quartiles [25%, 75%]	178.3 ; 304.7]	[171.6 ; 226.9]	[149.1 ; 196.2]
	PT (ms)	OD	Mean+/- SD	94.7 ± 7.4	99.4 ± 5.5	97.1 ± 9.0
			Median [min, max]	93.5 83.0 ; 112.5	100.0 91.5 ; 110.5	93.8 88.0 ; 121.5
			CoV %	7.8	5.6	9.3
			Percentile [5%, 95%]	[83.0 ; 106.5]	[91.5 ; 110.5]	[88.0 ; 121.5]
			Quartiles [25%, 75%]	[89.5 ; 99.3]	[95.5 ; 102.5]	[91.5 ; 100.5]
		OS	Mean+/- SD	94.1 ± 6.9	99.6 ± 6.0	97.4 ± 8.1
			Median [min, max]	94.8 81.9 ; 112.0	99.0 89.5 ; 115.5	95.0 87.0 ; 118.5
			CoV %	7.4	6.0	8.3
			Percentile [5%, 95%]	[83.0 ;107.5]	[89.5 ; 115.5]	[87.0 ; 118.5]
			Quartiles [25%, 75%]	[89.0 ; 97.8]	[96.5 ; 104.0]	[93.0 ; 101.3]

ERG Rod-cone response, DA 3.0 a-wave

		OD/OS	parameter	Site 1<br>Argentina	Site 2<br>Hungary	Site 3<br>Portugal
				N=28	N=18	N=16
				Age years<br>Mean+/- SD<br>63.8 ± 7.8	Age years<br>Mean+/- SD<br>62.9 ± 5.9	Age years<br>Mean+/- SD<br>64.9 ± 8.3
DA 3.0<br>a-wave	AMPL<br>(μV)	OD	Mean +/- SD	-191.8 ± 54.2	-165.4 ± 40.7	-164.0 ± 31.1
			Median<br>Min ;max	-178.5<br>-328.0 ; -105.4	-169.8<br>-248.1 ; -90.7	-167.7<br>-237.7 ; -99.3
			CoV M	-28.3	-24.6	-19.0
			Percentile [5%, 95%]	[-313.5 ; -119.8]	[-248.1 ; -90.7]	[-237.7 ; -99.3]
			Quartiles [25%, 75%]	[-223.9 ; -155.6]	[-192.4 ; -132.5]	[-174.9 ; -146.7]
		OS	Mean+/- SD	-197.6 ± 60.6	-168.6 ± 43.9	-163.3 ± 30.7
			Median<br>[min, max]	-191.6<br>-382.1 ; -120.5	-160.3<br>-273.3 ; -77.9	-167.1<br>-205.0 ; -107.8
			CoV %	-30.7	-26.0	-18.8
			Percentile [5%, 95%]	[-325.5 ; -122.9]	[-273.3; -77.9]	[-205.0 ; -107.8]
			Quartiles [25%, 75%]	[-210.6 ; -154.3]	[-200.2 ; -146.6]	[-190.3 ; -144.6]
	PT<br>(ms)	OD	Mean+/- SD	16.5 ± 0.8	16.8 ± 0.7	16.3 ± 1.0
			Median<br>[min, max]	16.5<br>15.0 ; 18.5	16.5<br>15.5 ; 18.0	16.0<br>14.5 ; 19.0
			CoV%	5.0	4.0	6.4
			Percentile [5%, 95%]	[15.5 ; 18.0]	[15.5 ;18.0]	[14.5 ; 19.0]
			Quartiles [25%, 75%]	[16.0 ; 17.0]	[16.5 ; 17.0]	[16.0 ; 16.8]
		OS	Mean+/- SD	16.4 ± 0.8	16.7 ± 0.6	16.3 ± 0.8
			Median<br>[min, max]	16.5<br>15.0 ; 18.5	16.8<br>16.0 ; 17.5	16.0<br>15.5 ; 18.0
			CoV%	5.0	3.4	4.8
			Percentile [5%, 95%]	[15.0 ; 17.5]	[16.0 ; 17.5]	[15.5 ; 18.0]
			Quartiles [25%, 75%]	[16.0 ; 17.0]	[16.0 ; 17.0]	[15.8 ; 17.0]

ERG Rod-cone response, DA 3.0 b-wave

		OD/OS	parameter	Site 1<br>Argentina	Site 2<br>Hungary	Site 3<br>Portugal
				N=28	N=18	N=16
				Age years	Age years	Age years

				Mean+/- SD<br>63.8 ± 7.8	Mean+/- SD<br>62.9 ± 5.9	Mean+/- SD<br>64.9 ± 8.3
DA<br>3 .0<br>b-wave	AMPL<br>(µV)	OD	Mean +/- SD	315.7 ± 77.4	301.0 ± 67.9	249.0 ± 48.7
			Median<br>Min ;max	326.5<br>163.0 ; 462.8	314.0<br>163.6 ; 372.2	230.4<br>185.6 ; 338.1
			CoV %	24.5	22.5	19.6
			Percentile [5%, 95%]	[211.6 ; 450.8]	[163.6 ;372.2]	[185.6 ; 338.1]
			Quartiles [25%, 75%]	[258.3 ; 358.5]	[245.3 ; 366.0]	[212.6 ; 281.4]
		OS	Mean+/- SD	321.9 ± 102.6	298.1 ± 80.2	248.6 ± 34.1
			Median<br>[min, max]	311.5<br>183.2 ; 626.1	287.5<br>162.9 ; 489.9	257.1<br>186.8 ; 291.3
			CoV %	31.9	26.9	13.7
			Percentile [5%, 95%]	[193.5 ; 528.8]	[162.9 ; 489.9]	[186.8 ; 291.3]
			Quartiles [25%, 75%]	[248.8 ; 371.8]	[246.0 ; 338.2]	[214.9 ; 279.4]
	PT<br>(ms)	OD	Mean+/- SD	54.1 ± 3.3	52.9 ± 2.4	53.7 ± 3.3
			Median<br>[min, max]	53.8<br>46.0 ; 60.5	53.0<br>48.5 ; 58.5	54.0<br>44.0 ; 58.5
			CoV%	6.2	4.5	6.2
			Percentile [5%, 95%]	[49.5 ; 60.0]	[48.5 ; 58.5]	[44.0 ; 58.5]
			Quartiles [25%, 75%]	[52.5 ; 56.0]	[51.5 ; 54.0]	[52.5 ; 56.3]
		OS	Mean+/- SD	54.1 ± 3.9	52.7 ± 2.1	53.9 ± 2.5
			Median<br>[min, max]	54.5<br>46.0 ; 63.0	52.3<br>49.5 ; 57.0	54.5<br>47.0 ; 56.9
			CoV%	7.1	4.0	4.7
			Percentile [5%, 95%]	[48.0 ; 60.0]	[49.5 ; 57.0)	[47.0 ; 56.9]
			Quartiles [25%, 75%]	[51.5 ; 56.0]	[51.0 ; 54.0]	[52.8 ; 55.8]

ERG Rod-cone response, DA 12.0 a-wave

		OD/OS	parameter	Site 1 Argentina	Site 2 Hungary	Site 3 Portugal
				N=28	N=18	N=16
				Age years Mean+/- SD 63.8 ± 7.8	Age years Mean+/- SD 62.9 ± 5.9	Age years Mean+/- SD 64.9 ± 8.1
DA 12.0 a- wave	AMPL (µV)	OD	Mean+/- SD	-222.8 ± 57.6	-207.4 ± 46.5	-186.8 ± 29.1
			Median [min, max]	-209.3 -380.0 ; -116.1	-210.5 -305.5 ; -126.5	-189.2 -257.3 ; -128.8
			CoV	-25.9	-22.4	-15.6
			Percentile [5%, 95%]	[-323.2 ; -149.4	[-305.5 ; -126.5]	[-257.3 ; -128.8]
			Quartiles [25%, 75%]	[-261.3 ; -184.2]	[-237.3 ; - 163.9]	[-195.6 ; -175.0]
		OS	Mean+/- SD	-231.7 ± 67.8	-212.9 ± 54.9	-189.5 ± 33.0
			Median [min, max]	-217.6 -431.9 ; -151.3	-206.5 -234.8 ; -181.5	-185.6 -243.5 ; -143.7
			CoV	-29.3	-25.8	-17.4
			Percentile [5%, 95%]	[-374.5 ; -156.1]	[-371.3 ;-120.0]	[-243.5 ; -143.7]
			Quartiles [25%, 75%]	[-250.1 ; -181.1]	[-234.8 ; -181.5]	[-212.4 ; -164.7]
	PT (ms)	OD	Mean+/- SD	13.1 ± 1.1	13.4 ± 0.8	12.3 ± 1.4
			Median [min, max]	13.0 11.0 ; 16.0	13.5 12.0 ; 15.0	11.8 10.5 ; 16.5
			CoV%	8.1	6.2	11.6
			Percentile [5%, 95%]	[11.5 ; 15.0)	[12.0 ;15.0]	[10.5 ; 16.5]
			Quartiles [25%, 75%]	[12.5 ; 13.5]	[13.0 ; 14.0]	[11.5 ; 13.0]
		OS	Mean+/- SD	13.1 ± 1.1	13.5 ± 0.9	12.4 ± 1.3
			Median [min, max]	13.0 11.5 ; 15.5	13.5 12.0 ; 15.0	12.0 11.0 ; 15.5
			CoV%	8.2	6.9	10.3
			Percentile [5%, 95%]	[12.0; 15.0]	[12.0 ; 15.0]	{11.0 ; 15.5]
			Quartiles [25%, 75%]	[12.3 ; 13.8]	[12.5 ; 14.5]	[11.5 ; 12.8]

ERG Rod-cone response, DA 12.0 b-wave

		OD/ OS	parameter	Site 1 Argentina	Site 2 Hungary	Site 3 Portugal
				N=28	N=18	N=16
				Age years Mean+/- SD	Age years Mean+/- SD	Age years Mean+/- SD

				63.8 ± 7.8	62.9 ± 5.9	64.9 ± 8.1
**DA 12.0 b-wave**	AMPL (μV)	OD	Mean+/- SD	319.1 ± 77.2	319.0 ± 66.9	255.8 ± 44.9
			Median [min, max]	313.8<br>144.0 ; 505.4	338.1<br>192.0 ; 398.1	235.2<br>198.5 ; 346.5
			CoV %	24.2	21.0	17.5
			Percentile [5%, 95%]	[212.5 ; 434.7]	[192.0 ; 398.1]	[198.5 ; 346.5]
			Quartiles [25%, 75%]	[265.1 ; 362.1]	[266.9 ; 382.3]	[221.7 ; 286.6]
		OS	Mean+/- SD	327.4 ± 103.3	317.0 ± 84.5	256.2 ± 30.8
			Median [min, max]	292.6<br>182.7 ; 630.1	308.9<br>184.3 ; 512.5	263.0<br>197.3 ; 298.2
			CoV %	31.6	26.7	12.0
			Percentile [5%, 95%]	[199.4 ; 533.8]	[184.3 ; 512.5]	[197.3 ; 298.2]
			Quartiles [25%, 75%]	[270.2 ; 366.6]	[252.8 ; 353.1]	[228.0 ; 279.3]
	PT (ms)	OD	Mean+/- SD	53.0 ± 4.6	52.2 ± 3.8	49.9 ± 4.6
			Median [min, max]	53.0<br>44.4 ; 66.5	52.8<br>41.5 ; 60.0	51.3<br>41.0 ; 59.0
			CoV%	8.6	7.4	9.3
			Percentile [5%, 95%]	[45.5 ; 60.0]	[41.5 ; 60.0]	[41.0 ; 59.0]
			Quartiles [25%, 75%]	[50.5 ; 55.0]	[50.0 ; 55.0]	[45.5 ; 52.3]
		OS	Mean+/- SD	52.1 ± 4.6	51.6 ± 4.1	50.4 ± 4.3
			Median [min, max]	52.0<br>42.5 ; 64.5	51.5<br>42.0 ; 60.0	51.8<br>42.5 ; 56.5
			CoV%	8.7	8.0	8.5
			Percentile [5%, 95%]	[45.5 ; 62.0]	[42.0 ; 60.0]	[42.5 ; 56.5]
			Quartiles [25%, 75%]	[49.0 ; 55.0]	[49.5 ; 55.0]	[47.5 ; 53.6]

ERG single flash cone response, LA 3.0 a-wave

		OD/OS	parameter	Site 1<br>Argentina	Site 2<br>Hungary	Site 3<br>Portugal
				N=28	N=18	N=16
				Age years<br>Mean+/- SD<br>63.8 ± 7.8	Age years<br>Mean+/- SD<br>62.9 ± 5.9	Age years<br>Mean+/- SD<br>64.9 ± 8.3
LA 3.0<br>a-wave	AMPL<br>(µV)	OD	Mean+/- SD	-32.2 ± 9.4	-26.9 ± 6.6	-22.0 ± 4.3
			Median<br>[min, max]	-31.6<br>-50.3 ; -16.4	-28.1<br>-38.1 ; -12.3	-23.0<br>-28.5 ; -15.4
			CoV %	-29.3	-24.5	-19.3
			Percentile [5%, 95%]	[-46.4 ; -17.1]	[-38.1 ; -12.3]	[-28.5 ; -15.4]
			Quartiles [25%, 75%]	[-40.2 ; -26.0]	[-31.7 ; -21.8]	[-24.6 ; -18.6]
		OS	Mean+/- SD	-34.3 ± 12.4	-27.8 ± 5.0	-22.5 ± 5.5
			Median<br>[min, max]	-30.3<br>-62.7 ; -17.0	-28.3<br>-38.0 ; -13.2	-22.2<br>-33.9 ; -14.5
			CoV %	-36.1	-18.1	-24.5
			Percentile [5%, 95%]	[-59.1 ; -17.6]	[-38.0 ; -13.2]	[-33.9 ; -14.5]
			Quartiles [25%, 75%]	[-39.9 ; -26.3]	[-29.9 ; -26.7]	[-26.9 ; -17.6]
	PT<br>(ms)	OD	Mean+/- SD	15.3 ± 0.8	15.2 ± 0.5	14.7 ± 0.9
			Median<br>[min, max]	15.0<br>14.5 ; 17.5	15.0<br>14.0 ; 16.5	14.8<br>13.0 ; 17.0
			CoV %	5.1	3.6	6.1
			Percentile [5%, 95%]	[14.5 ; 16.5]	[14.0 ; 16.5]	[13.0 ; 17.0]
			Quartiles [25%, 75%]	[14.5 ; 15.8]	[15.0 ; 15.5]	[14.3 ; 15.0]
		OS	Mean+/- SD	15.1 ± 0.9	15.3 ± 0.6	14.8 ± 1.0
			Median<br>[min, max]	15.2<br>13.0 ; 16.5	15.3<br>14.0 ; 16.5	14.5<br>13.0 ; 17.0
			CoV%	5.8	4.2	6.7
			Percentile [5%, 95%]	[13.5 ; 16.5]	[14.0 ; 16.5]	[13.0 ; 17.0]
			Quartiles [25%, 75%]	[14.5 ; 15.5]	[15.0 ; 16.0]	[14.5 ; 15.0

ERG single flash cone response, LA 3.0 b-wave

		OD/OS	parameter	Site 1<br>Argentina	Site 2<br>Hungary	Site 3<br>Portugal
				N=28	N=18	N=16
				Age years<br>Mean+/- SD	Age years<br>Mean+/- SD	Age years<br>Mean+/- SD

				63.8 ± 7.8	62.9 ± 5.9	64.9 ± 8.3
LA 3.0 b-wave	AMPL (µV)	OD	Mean+/- SD	128.0 ± 27.0	111.4 ± 23.8	93.4 ± 22.1
			Median [min, max]	127.2<br>89.4 ; 190.1	120.8<br>50.2 ; 137.1	97.4<br>61.6 ; 132.3
			CoV %	21.1	21.4	23.7
			Percentile [5%, 95%]	[91.7 ; 173.4]	[50.2 ; 137.1]	[61.6 ; 132.3]
			Quartiles [25%, 75%]	[104.8 ; 143.6]	[91.0 ; 130.4]	[69.2 ; 107.4]
		OS	Mean+/- SD	132.4 ± 33.7	111.4 ± 26.7	94.3 ± 22.8
			Median [min, max]	123.2<br>76.8 ; 225.3	109.6<br>42.6 ; 168.4	98.6<br>52.0 ; 130.4
			CoV %	25.5	24.0	24.1
			Percentile [5%, 95%]	[95.6 ; 204.9]	[42.6 ; 168.4]	[52.0 ; 130.4]
			Quartiles [25%, 75%]	[112.6 ; 148.0]	[101.0 ; 123.6]	[75.3 ; 105.4]
	PT (ms)	OD	Mean+/- SD	31.6 ± 1.4	30.1 ± 1.1	31.1 ± 1.0
			Median [min, max]	31.5<br>28.0 ; 35.0	30.0<br>28.5 ; 33.0	30.8<br>30.0 ; 33.0
			CoV%	4.4	3.8	3.3
			Percentile [5%, 95%]	[30.0 ; 33.5]	[28.5 ; 33.0]	[30.0 ; 33.0]
			Quartiles [25%, 75%]	[30.5 ; 32.5]	[29.0 ; 31.0]	[30.5 ; 32.3]
		OS	Mean+/- SD	31.4 ± 1.2	30.1 ± 1.3	31.2 ± 1.2
			Median [min, max]	31.5<br>28.0 ; 34.0	29.8<br>28.0 ; 33.0	31.0<br>29.5 ; 33.5
			CoV%	3.9	4.2	3.7
			Percentile [5%, 95%]	[29.0 ; 33.0]	[28.0 ; 33.0]	[29.5 ; 33.5]
			Quartiles [25%, 75%]	[30.5 ; 32.3]	[29.0 ; 31.0]	[30.3 ; 32.0]

ERG flicker response, LA 3.0 30 Hz

		OD/OS	parameter	Site 1 Argentina	Site 2 Hungary	Site 3 Portugal
				N=28	N=18	N=16
				Age years Mean+/- SD 63.8 ± 7.8	Age years Mean+/- SD 62.9 ± 5.9	Age years Mean+/- SD 64.9 ± 8.3
LA 3.0 Flicker 30 Hz	AMPL (µV)	OD	Mean+/- SD	103.6 ± 23.4	85.9 ± 22.1	73.9 ± 15.9
			Median [min, max]	97.9 59.5 ; 165.0	93.7 32.1 ; 116.1	74.6 40.9 ; 97.8
			CoV %	22.6	25.8	21.6
			Percentile [5%, 95%]	[75.4 ; 146.3]	[32.1 ; 116.1]	[40.9 ; 97.8]
			Quartiles [25%, 75%]	[88.5 ; 117.1]	[66.0 ; 100.2]	[62.7 ; 88.1]
		OS	Mean+/- SD	105.6 ± 24.2	86.8 ± 24.6	75.4 ± 15.0
			Median [min, max]	103.9 63.9 ; 188.7	84.8 25.7 ; 140.2	75.8 51.3 ; 104.7
			CoV %	22.9	28.3	19.9
			Percentile [5%, 95%]	[66.2 146.9]	[25.7 ; 140.2]	[51.3 ; 104.7]
			Quartiles [25%, 75%]	[93.4 ; 115.0]	[77.7 ; 99.9]	[62.8 ; 87.5]
	PT (ms)	OD	Mean+/- SD	28.9 ± 1.6	26.9 ± 1.5	28.0 ± 1.2
			Median [min, max]	29.0 25.5 ; 33.0	26.5 25.0 ; 31.5	28.0 26.0 ; 30.5
			CoV%	5.5	5.5	4.2
			Percentile [5%, 95%]	[27.0 ; 31.5]	[25.0; 31.5]	[26.0 ; 30.5]
			Quartiles [25%, 75%]	[27.8 ; 30.0]	[26.0 ; 27.5]	[27.0 ; 28.8]
		OS	Mean+/- SD	28.7 ± 1.6	27.1 ± 1.6	28.2 ± 1.3
			Median [min, max]	28.8 25.5 ; 32.0	27.0 24.5 ; 31.0	28.5 25.5 ; 31.0
			CoV%	5.7	5.7	4.6
			Percentile [5%, 95%]	[26.5 ; 32.0]	[24.5 ; 31.0]	[25.5 ; 31.0]
			Quartiles [25%, 75%]	[27.3 ; 30.0]	[26.0 ; 27.5]	[27.0 ; 29.0]

Best Corrected Visual Acuity (BCVA)

		OD/OS	parameter	Site 1 Argentina	Site 2 Hungary	Site 3 Portugal
				N=28	N=18	N=16
				Age years Mean+/- SD 63.8 ± 7.8	Age years Mean+/- SD 62.9 ± 5.9	Age years Mean+/- SD 64.9 ± 8.3
BCVA	Log MAR	OD	Mean+/- SD	0.00714 ± 0.02623	-0.01667 ± 0.05145	-0.03750 ± 0.08062
			Median [min, max]	0.00000 0.0 ; 0.1	0.00000 -0.1 ; 0.1	-0.10000 -0.1 ; 0.1
			CoV%	367.17137	-308.69745	-214.99354
			Percentile [5%, 95%]	[0.00 ; 0.10]	[-0.10 ; 0.10]	[-0.10 ; 0.10]
			Quartiles [25%, 75%]	[0.00000 ; 0.00000]	[0.00000 ; 0.00000]	[-0.10000 ; 0.00000]
		OS	Mean+/- SD	0.00714 ± 0.03780	-0.00556 ± 0.08726	0.00000 ± 0.11547
			Median [min, max]	0.00000 0.0 ; 0.2	0.00000 -0.1 ; 0.2	-0.05000 -0.1 ; 0.2
			CoV%	529.15026	-1570.6874	
			Percentile [5%, 95%]	[0.00 0.00]	[-0.10 ; 0.20]	[-0.10 ; 0.20]
			Quartiles [25%, 75%]	[0.00000 ; 0.00000]	[-0.10000 ; 0.00000]	[-0.10000 ; 0.10000]

Color vision (CV)

		OD/OS	parameter	Site 1 Argentina	Site 2 Hungary	Site 3 Portugal
				N=28	N=18	N=16
				Age years Mean+/- SD 63.8 ± 7.8	Age years Mean+/- SD 62.9 ± 5.9	Age years Mean+/- SD 64.9 ± 8.3
CV	TES	OD	Mean+/- SD	51.8 ± 41.5	41.2 ± 37.7	26.9 ± 33.2
			Median [min, max]	46.0 0.0 ; 142.0	38.0 0.0 ; 112.0	14.0 0.0 ; 88.0
			CoV%	80.2	91.4	123.5
			Percentile [5%, 95%]	[0.0 ; 130.0]	[0.0 ;112.0]	[0.0 ; 88.0]
			Quartiles [25%, 75%]	[16.0 ; 72.0]	[0.0 ; 64.0]	[0.0 ; 49.0]
		OS	Mean+/- SD	56.2 ± 42.5	46.2 ± 46.2	25.6 ± 26.8
			Median [min, max]	52.0 0.0 ; 146.0	28.0 0.0 ; 138.0	24.0 0.0 ; 114.0
			CoV %	75.7	100.0	104.7
			Percentile [5%, 95%]	[8.0 ; 142.0]	[0.0 ; 138.0]	[0.0 ; 114.0]
			Quartiles [25%, 75%]	[16.0 ; 93.0]	[0.0 ; 88.0]	[12.0 ; 32.0]

VF static

Test		OD/OS	parameter	Site 1<br>Argentina	Site 2<br>Hungary	Site 3<br>Portugal
				N=28	N=18	N=16
				Age years<br>Mean+/- SD<br>63.8 ± 7.8	Age years<br>Mean+/- SD<br>62.9 ± 5.9	Age years<br>Mean+/- SD<br>64.9 ± 8.3
VFs	MD (db)	OD	Mean+/- SD	1.5 ± 1.9	1.3 ± 1.8	2.8 ± 3.8
			Median<br>[min, max]	1.3<br>-1.5 ; 6.6	1.1<br>-2.9 ; 4.1	1.6<br>-0.3 ; 14.8
			CoV	121.8	141.5	138.1
			Percentile [5%, 95%]	[-1.0 ; 5.8 }	[-2.9; 4.1]	[-0.3 ; 14.8]
			Quartiles [25%, 75%]	[0.4 ; 2.1 }	[0.3 ; 2.5]	[0.8 ; 3.3]
		OS	Mean+/- SD	2.0 ± 2.3	2.2 ± 2.9	3.0 ± 2.9
			Median<br>[min, max]	1.6<br>-0.7 ; 9.3	1.8<br>-0.8 ; 11.5	2.5<br>-0.6 ; 9.7
			CoV	116.6	132.4	99.9
			Percentile [5%, 95%]	[-0.4 ; 7.1]	[-0.8 ; 11.5]	[-0.6 ;9.7}
			Quartiles [25%, 75%]	[0.5 ; 2.3]	[0.0 ; 2.9]	[0.8 ; 4.3

VF Kinetic

	Isopter	OD/OS	parameter	Site 1 Argentina	Site 2 Hungary	Site 3 Portugal
				N=28	N=18	N=16
				Age years Mean+/- SD 63.8 ± 7.8	Age years Mean+/- SD 62.9 ± 5.9	Age years Mean+/- SD 64.9 ± 8.3
VFk	I3e Area (deg2)	OD	Mean+/- SD	6504.8 ± 1165.4	6363.2 ± 1075.3	5418.1 ± 1179.5
			Median [min, max]	6625.5 4197.0 ; 9661.0	6353.5 4000.0 ; 8637.0	5439.5 2930.0 ; 6976.0
			CoV %	17.9	16.9	21.8
			Percentile [5%, 95%]	[4668.0; 8940.0]	[4000.0; 8637.0]	[2930.0 ; 6976.0]
			Quartiles [25%, 75%]	[5666.5 ; 7060.0]	[5859.0 ; 6752.0]	[4648.5 ; 6564.5]
		OS	Mean+/- SD	6293.3 ± 1354.7	6732.7 ± 955.4	5486.9 ± 1484.9
			Median [min, max]	5959.5 4101.0 ; 9541.0	6770.5 4990.0 ; 8369.0	5374.5 2530.0 ; 7932.0
			CoV %	21.5	14.2	27.1
			Percentile [5%, 95%]	[4421.0 8515.0]	[4990.0 ; 8369.0]	[2530.0; 7932.0]
			Quartiles [25%, 75%]	[5327.5 ; 7143.5]	[6110.0 ; 7455.0]	[4651.5 ; 6707.5]
	III4e Area (deg2)	OD	Mean+/- SD	13165.9 ± 1411.7	14087.2 ± 1265.4	12524.9 ± 1828.2
			Median [min, max]	13483.0 10402.0 ; 16022.0	14160.5 12021.0 ; 16471.0	12304.5 8606.0 ; 15479.0
			CoV%	10.7	9.0	14.6
			Percentile [5%, 95%]	[10501.0 ; 15217.0]	[12021.0 ; 16471.0]	[8606.0 ; 15479.0
			Quartiles [25%, 75%]	[12317.0 ; 14165.5]	[13114.0 ; 14769.0]	[11714.5 ; 14137]0
		OS	Mean+/- SD	13436.9 ± 1290.6	14031.3 ± 1563.3	12520.3 ± 1567.6
			Median [min, max]	13494.0 9909.0 ; 15594.0	14267.5 9681.0 ; 16482.0	11939.0 10277.0 ; 15400.0
			CoV %	9.6	11.1	12.5
			Percentile [5%, 95%]	[11414.0 ; 15249.0]	[9681.0 ; 16482.0]	[10277.0 ; 15400.0]
			Quartiles [25%, 75%]	[12621.5 ; 14459.0]	[13655.0 ; 14985.0]	[11660.0 ; 13765.5]
